# Charge-Transfer State Dissociation Efficiency Can Limit Free Charge Generation in Low-Offset Organic Solar Cells

**DOI:** 10.1021/acsenergylett.3c00943

**Published:** 2023-07-14

**Authors:** Jolanda Simone Müller, Marc Comí, Flurin Eisner, Mohammed Azzouzi, Diego Herrera Ruiz, Jun Yan, Salahuddin Sayedshabbir Attar, Mohammed Al-Hashimi, Jenny Nelson

**Affiliations:** † Department of Physics and Centre for processable Electronics, Imperial College London, Blackett Laboratory, Prince Consort Road, London SW7 2AZ, United Kingdom; ‡ Department of Arts and Sciences, Texas A&M University at Qatar, Education City, P.O. Box 23874, Doha, Qatar; ∥ School of Science and Engineering, The Chinese University of Hong Kong, Shenzhen, Guangdong Province 518172, P. R. China

## Abstract

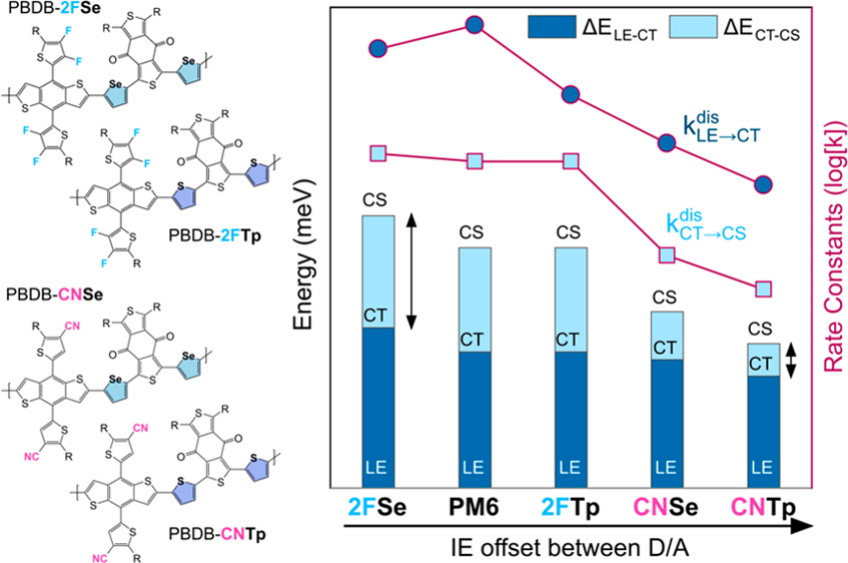

We investigate the charge-generation processes limiting the performance of low-offset organic bulk-heterojunction solar cells by studying a series of newly synthesized PBDB-T-derivative donor polymers whose ionisation energy (IE) is tuned via functional group (difluorination or cyanation) and backbone (thiophene or selenophene bridge) modifications. When blended with the acceptor Y6, the series present heterojunction donor–acceptor IE offsets (*ΔE*_IE_) ranging from 0.22 to 0.59 eV. As expected, small *ΔE*_IE_ decrease nonradiative voltage losses but severely suppresses photocurrent generation. We explore the origin of this reduced charge-generation efficiency at low *ΔE*_IE_ through a combination of opto-electronic and spectroscopic measurements and molecular and device-level modeling. We find that, in addition to the expected decrease in local exciton dissociation efficiency, reducing *ΔE*_IE_ also strongly reduces the charge transfer (CT) state dissociation efficiency, demonstrating that poor CT-state dissociation can limit the performance of low-offset heterojunction solar cells.

Organic photovoltaics (OPVs) are of great interest for renewable energy production due to their rapid energy payback, low ecotoxicity, and high tunability of optical and energetic properties, making them employable in a large range of applications.^1−4^ The power conversion efficiencies (PCEs) of bulk-heterojunction (BHJ) solar cells are approaching 20%.^5,6^ This has been achieved thanks to improvements in the chemical design of the donor (D) and acceptor (A) components and the introduction of blends with a low offset between D and A ionization energies (IE) using small-molecule nonfullerene acceptors (NFAs) such as ITIC or Y6.^7,8^ Systems with low IE offsets (*ΔE*_IE_) have gained a lot of interest due to their potential to reduce nonradiative voltage losses^9−12^ to maximize the open-circuit voltage (*V*_oc_),^13^ however, offsets that are too small tend to limit the charge-generation efficiency.^14,15^ So far, charge generation in OPVs following the photogeneration of an exciton is commonly accepted to be a two-stage process: 1) the formation of a charge-transfer (CT) exciton at the donor–acceptor interface, at a rate *k*_LE→CT_^dis^; 2) the dissociation of the CT exciton into a charge-separated (CS) state (free charges) with efficiency *k*_CT→CS_^dis^. The overall charge-generation (*k*_LE→CS_^dis^) efficiency thus requires both efficient *k*_LE→CT_^dis^ and *k*_CT→CS_^dis^. Recent work has shown that a minimum IE offset of 0.5 eV is required for both processes to proceed efficiently.^15^ However, it is still under debate how changing *ΔE*_IE_ separately affects *k*_LE→CT_^dis^ and *k*_LE→CS_^dis^ in different material systems^16−21^ and how this affects obtainable voltage losses and charge-generation efficiencies. It has been particularly challenging to determine the energies of the CT and CS states, because the CT state often lies very close to the brighter local exciton (LE) state in low-offset systems,^22,23^ and there is no definite relationship between the CS energy in bulk blends and measurable related quantities like *ΔE*_IE_ of pristine materials. A greater understanding of these energetics would help design materials and structures that can potentially overcome this limit.

Strategies to tune the IE levels in donor polymers include the addition of stronger electron withdrawing moieties that lower the electron density in the conjugated backbone to deepen (i.e., shift further from vacuum) the IE.^24−26^ In the popular donor polymer PBDB-T,^27^ this has been achieved, for example, through chlorination,^28^ the addition of alkylthiol substituents,^29^ or fluorination^30^ to form structures such as PBDB-TCl, PBDB-TSF, or PBDB-TF (PM6),^31^ affording PCEs of up to 19.3%, and nonradiative voltage losses approaching 0.2 eV.^6,32,33^ On the other hand, small upward shifts in the IE (shallower; closer to vacuum) have been achieved in different donor polymers when replacing the thiophene unit with a selenophene moiety.^31,34−36^

Herein, we explore the factors limiting charge generation in low offset systems, by tuning the IE offsets between components in the polymer-nonfullerene OPV devices using electron withdrawing groups and selenophene substitution, obtaining a range of offsets from 0.22 to 0.59 eV. We lower the energy levels of the polymer by adding two different electron withdrawing functional groups (difluoro (2F) and nitrile (CN)) at the 3,4-position of the thiophene substituents on the BDT monomer and fine-tune the IE (and narrow the bandgap) with backbone modifications replacing the spacer thiophene (Tp) unit with selenophene (Se) for the BDTD monomer. Using each possible combination of the suggested substitutions, we synthesized four PBDB-T derivatives that contain PBDB-2F-Tp (abbreviated 2FTp henceforth), PBDB-CN-Tp (CNTp), PBDB-2F-Se (2FSe), and PBDB-CN-Se (CNSe). We analyze the effects of these substitutions on the device performance and charge-generation efficiency using electroluminescence (EL), field- and light-bias dependent external quantum efficiency (EQE), and space-charge-limited-current (SCLC) measurements. From this, we explore the correlation between the D/A offset *ΔE*_IE_ and the device performance metrics. While finding the expected increase of the *V*_oc_ for the lower IE offsets, the low offsets coincide with compromised charge separation and increased bimolecular recombination. By fitting these results with a comprehensive model that combines charge-transfer processes with a semiconductor device model, we explain the impact of the low offset in terms of properties of the states involved in the photogeneration process, such as dissociation rates of excitons into the CT state *k*_LE→CT_^dis^ and from CT to the CS state *k*_LE→CS_^dis^. We find that CT state dissociation can be a limiting factor for efficient charge generation in systems with an offset between donor and acceptor ionization potentials of below 0.3 eV.

## Materials Analysis

**Figure 1:**
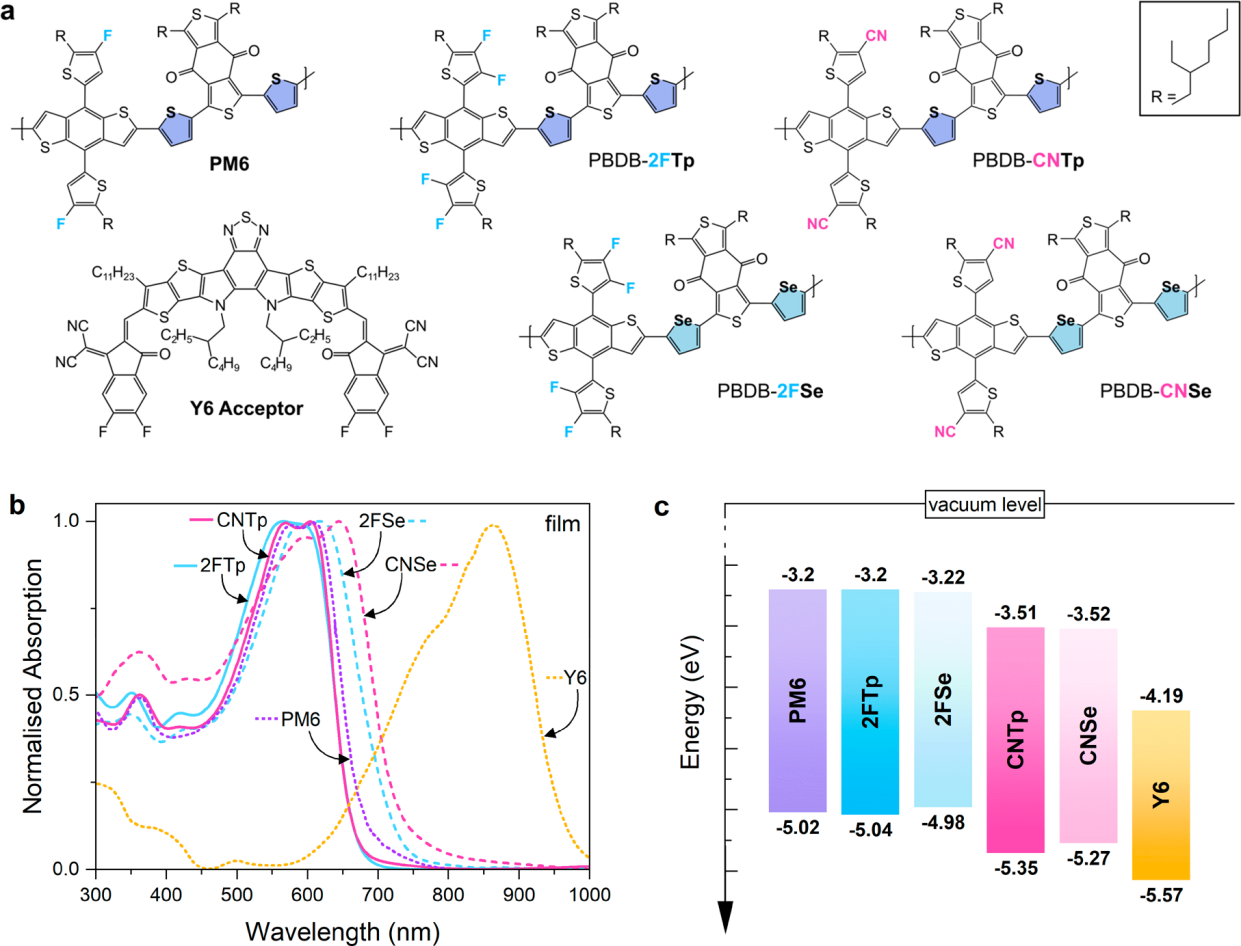
(a) Chemical structures of the donor polymers investigated in the study, with the systematic substitution of heteroatoms and functional groups indicated. The chemical structure of Y6, which was used as acceptor, is also shown. (b) UV–vis absorption spectra of thin films of the polymers and Y6. PM6 absorption data taken from Zhang et al.^30^ (c) The energy levels of the materials used in this study. Ionization energies (approximating the HOMO energy level) were obtained from APS^39^ (Figure S4) and the LUMO energies were calculated using the optical bandgap energies retrieved from the intersection between the photoluminescence of the pristine materials (Figure S5) and the absorbance,^40^ as shown in Figure S6.

The chemical structures of the novel synthesized polymers are shown in Figure 1a alongside the structure of PM6, which we use as a reference polymer, and that of Y6,^37^ which we use as the acceptor in blends. Details about the synthesis are provided in the Supporting Information (Section 1, Figure S1 and Figure S2). The measured UV–vis absorption spectra of the donor polymers and the Y6 acceptor molecule thin films are shown in Figure 1b (and in solution in Figure S3). In agreement with previous work,^38^ the donor polymers containing the selenophene unit (2FSe and CNSe) show a red shift in the absorption edge compared to those with the thiophene moiety, indicating a narrower optical bandgap, while there is little effect of functional group modification on absorption characteristics. In Figure 1c, we show the IE—approximating the highest occupied molecular orbital (HOMO)—of the materials obtained from ambient pressure photoemission spectroscopy (APS) measurements^39^ (Figure S4) and the lowest unoccupied molecular orbital (LUMO) energies, estimated by adding the optical band gap energy (Figures S5 and S6) to the measured IE. Interestingly, we observe that the extra fluorination of 2FTp compared to PM6 does not induce a lowering of energy levels. By contrast, introducing the nitrile substitution significantly lowers both the HOMO and the LUMO levels of the polymer compared to the fluorine substituent. This effect can be assigned to the stronger electron withdrawing properties of the nitrile (−CN) group compared to the fluorine (−F) group. In addition, it appears that the narrowing of the bandgap upon the substitution of the thiophene unit with selenophene (Figure 1b) results to a larger part from a lowering of the LUMO, rather than an increase in the HOMO energy level. This is consistent with previous research linking the lower bandgap in structurally similar selenophene-bridge copolymers to a stronger quinoidal character due to the lower aromatic stabilization energy in comparison to thiophene-bridged copolymers.^34^ We note that the experimentally measured trends in the energy levels and bandgap were confirmed qualitatively by density functional theory (DFT) calculations on the polymer monomers (Figure S7, Figure S8). The computational analysis also suggests that the selenophene substitution leads to a small change in the dihedral angles around the selenophene bridge, but overall, no flattening of the backbone is observed (Figure S9). We use the energy levels in neat films as an approximation for their values in blend, since they were found to be similar in PM6:Y6 in recent literature.^18^

## Photovoltaic Device Performance

As shown in Figure 1c, pairing the four newly synthesized polymers, along with PM6, with Y6 enables the fabrication of blends with systemically tuned offsets in the D/A *ΔE*_IE_, ranging from 0.59 eV (2FSe/Y6) to 0.22 eV (CNTp/Y6). To investigate how the changes in chemical structure of the donor polymer, and the concurrent changes in *ΔE*_IE_, affect the performance of a BHJ solar cell, we fabricated normal-architecture solar cell devices using poly(3,4-ethylenedioxythiophene)polystyrenesulfonate (PEDOT:PSS) as the hole transport layer and poly(9,9-bis(3′-(*N*,*N*-dimethyl)-*N*-ethylammoinium-propyl-2,7-fluorene)-*alt*-2,7-(9,9-dioctylfluorene))dibromide (PFN-Br) as the electron transport layer using the following architecture: glass substrate coated with indium tin oxide (ITO)/PEDOT:PSS/Donor:Acceptor/PFN-Br/Ag, where the donor:acceptor (D:A) blend is a mixture of one of the four PBDB-T derivative polymers (or the PM6 reference) and Y6. All blends were processed from chloroform with 0.5% chloronaphthalene additive and a D/A ratio of 1:1.2, which produced the best performance for all materials (Fabrication Details in the Supporting Information, Section 2). The polymer-in-solvent weight concentration was first tuned for performance (highest PCE devices) and then to obtain comparable thicknesses (same-thickness devices). The best performing devices for each blend are presented in Table 1 with the corresponding current–voltage characteristics shown in Figure S10. Under illumination, 2FTp shows the best performance, with a PCE of 14.3%, somewhat lower than that of the PM6 reference (16.0%) due to a lower fill factor (FF).

**Table 1:** Performance Values of Devices with Similar Thickness Active Layers and of the Best Device Made from Each Material (Indicated by a Star)^a^

	Device	Thickness (nm)	*V*_oc_ (V)	*J*_sc_*J*–*V*(mA/cm^2^)	*J*_sc_ EQE(mA/cm^2^)	FF (%)	PCE (%)
Same Thickness	**2FSe:Y6**	90 ± 10	0.792 ± 0.003	25.5 ± 0.6	24.5	46 ± 1	9.3 ± 0.3
	**2FTp:Y6**	110 ± 10	0.837 ± 0.003	26.6 ± 1.0	25.6	51 ± 2	11.5 ± 0.3
	**CNSe:Y6**	95 ± 10	0.896 ± 0.004	13.0 ± 0.4	12.5	40 ± 1	4.6 ± 0.1
	**CNTp:Y6**	100 ± 10	0.922 ± 0.011	7.3 ± 0.4	6.1	34 ± 1	2.3 ± 0.1
Best Device	2FSe:Y6*	50 ± 10	0.81	23.5		66	12.5
	2FTp:Y6*	85 ± 10	0.85	27.6		61	14.3
	CNSe:Y6*	95 ± 10	0.89	13.1		42	4.9
	CNTp:Y6*	80 ± 10	0.94	9.0		36	3.0
Reference	**PM6:Y6**	90 ± 10	0.838 ± 0.005	27.9 ± 1.1		68 ± 1	16.0 ± 0.7

a Same thickness devices were averaged over at least 17 devices.

**Figure 2:**
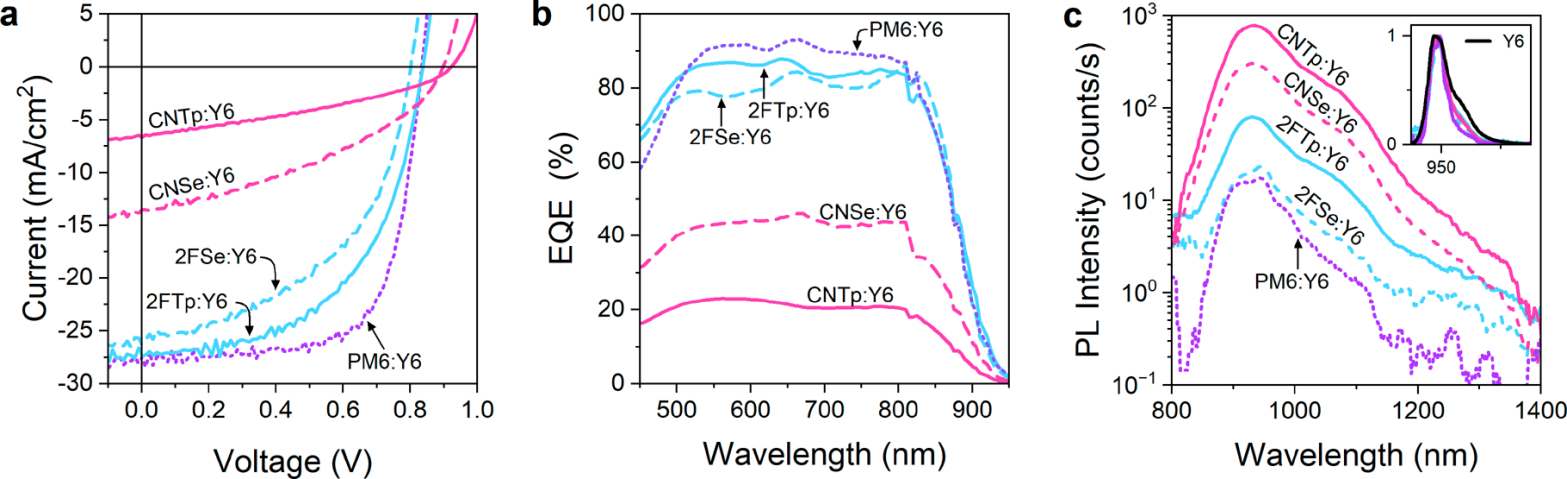
Performance characterization of the PBDBT-derivate polymer:Y6 devices of similar active layer thickness. (a) Current–voltage (*JV*) characteristics under one sun illumination (EM1.5) (b) EQE spectra. (c) Photoluminescence (PL) spectra of devices at open circuit under laser illumination (473 nm, 2 mW) normalized with the absorption at the excitation wavelength. Both EL and PL measurements were taken with an integration time of 10 s for CNTp and 30 s for the rest and are normalized over the integration time. While the emission does not represent a total photon count (no integrating sphere was used), the values are comparable relative to each other since they were measured under the same conditions for all materials. The inset shows the PL intensity of the devices normalized to the maximum alongside the PL emission of pristine Y6. PL of the pristine materials (polymers and Y6) is also shown in Figure S5.

In the proceding sections, we will focus on the devices with comparable thicknesses (90–110 nm) and processing conditions to maintain consistency and comparability across the results. The device performance parameters under one-sun (AM1.5) illumination are shown in Table 1, with current–voltage (*JV*) curves of corresponding devices shown in Figure 2a. Corresponding *JV* measurements in the dark are shown in Figure S11.

The PCE in the devices with similar thicknesses follows the same trend that is seen in the best performing devices. The same-thickness devices show an overall drop in the FF compared to the best performing ones, but all of the *J*_sc_ and *V*_oc_ values are similar. The trend in *V*_oc_ for all materials follows the expected trend according to the experimentally and theoretically measured energy levels, with deeper IE levels (and lower *ΔE*_IE_) leading to increased *V*_oc_. The addition of the second fluorine in 2FTp does not change the *V*_oc_ compared to PM6, but cyanation leads to a large increase in *V*_oc_ of around 0.1 V, while selenophene substitution introduced a small reduction in *V*_oc_ (0.03–0.04 V). The measured *J*_sc_ values are significantly higher in the fluorinated unit than in the cyanated polymer devices, in agreement with their higher driving force for charge separation due to their larger *ΔE*_IE_. However, the impact of selenophene substitution on *J*_sc_ is not consistent across both types of polymers: while it has only a minimal effect on *J*_sc_ in the fluorinated polymers, the substitution leads to a considerable increase in *J*_sc_ in the cyanated polymers. Finally, the FF are much higher in the 2F devices compared to the CN devices, while there is no clear dependence of FF on Se substitution. The PCE shows the same trends we see in the FFs: The fluorinated polymer devices show a much higher PCE than the CN devices, while the Se substitution decreases the cell performance in the former and improves it in the latter case.

To determine the spectral dependence of the charge generation, EQE spectra of the devices were measured (Figure 2b). The trends in short-circuit photocurrent measurements are replicated in the magnitude of EQE spectral response of the devices (i.e., a positive correlation between *ΔE*_IE_ and EQE magnitude), which further show no significant changes in the spectral shape between the different polymer blend devices. The *J*_sc_ values calculated from integrated EQE spectra confirm the *J*_sc_ trend from the AM1.5 *JV* measurement (Table 1). In order to determine whether the trends in charge-generation efficiency are primarily charge-collection or charge-separation related, we performed photoluminescence (PL) measurement on the blend devices at open circuit, with the results shown in Figure 2c. The spectral shape of the PL is similar to that of pristine Y6, which indicates that we are probing the emission of Y6 excited states. The trend in PL intensity is in direct anticorrelation with maximum EQE (higher EQE correlates to lower PL intensity). We further find a stronger voltage dependent PL peak intensity reduction in only the CN devices, indicating that poor exciton dissociation efficiency plays a role in the low offset systems (Figure S13). The PL spectra are discussed in more detail below, together with the EL emission spectra.

## Charge Separation and Recombination

**Figure 3:**
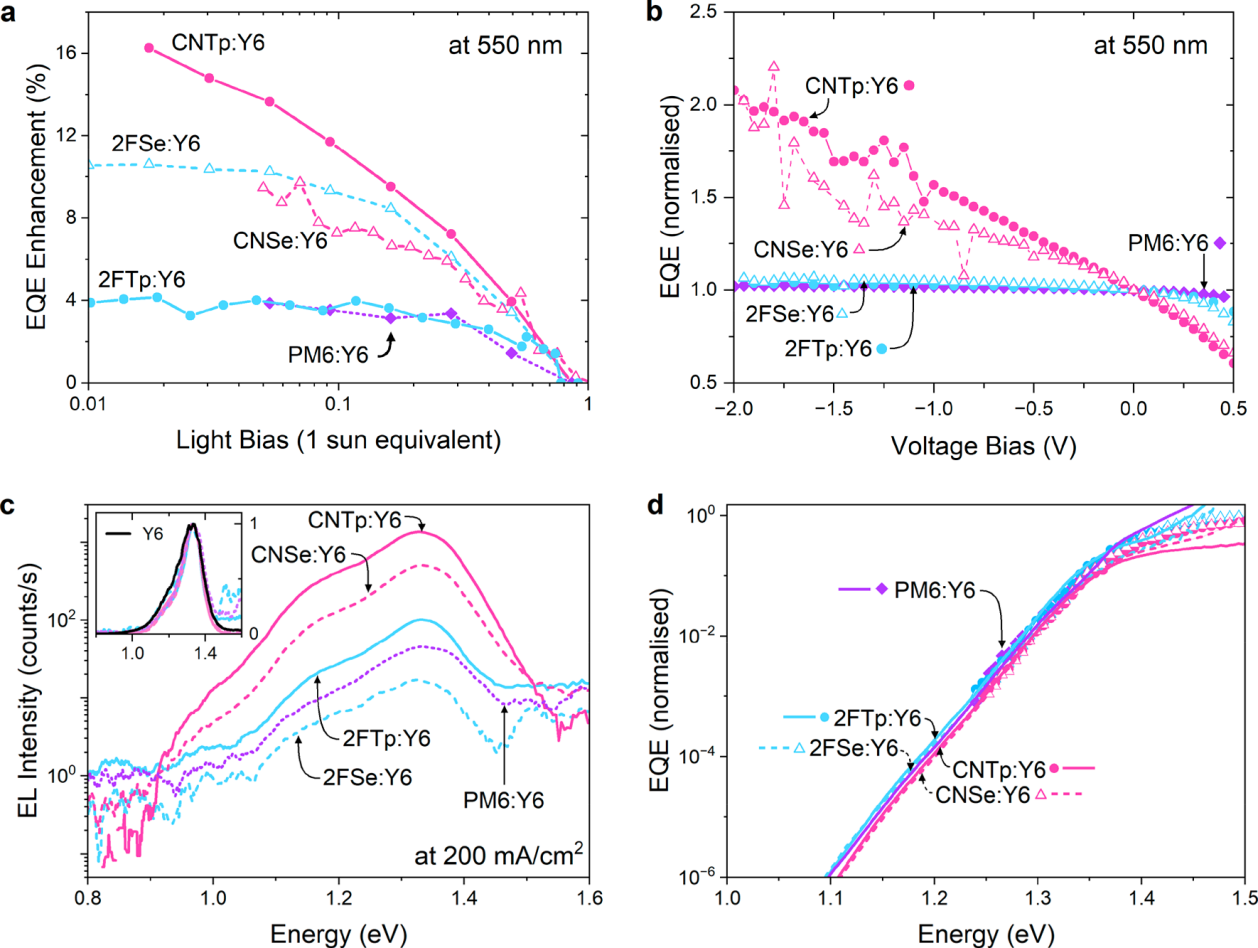
Electro-optical characterization of the PBDBT-derivate polymer:Y6 devices. Both EQE measurements are performed with the same light source at a fixed illumination wavelength of 550 nm, such that the absolute EQE values at 1 sun and zero-voltage are the same. (a) Light-bias dependent EQE, normalized to the value at 1 sun equivalent. (b) Voltage-bias dependent EQE at low fluence (see Figure S17), normalized to the value at zero bias. (c) Electroluminescence (EL) spectra measured at a 200 mA cm^–2^ injection current. The data are normalized over the integration time. The inset shows the EL data normalized to the peak value comparing the emission of the blends and the Y6 acceptor alone. (d) EQE data extended to lower values with the EL spectra by using the reciprocity relation. These data suggest a similar Urbach tail energy for all five materials of approximately 22 meV.

In order to gain additional understanding about how the processes governing charge generation in these devices are influenced by the offset *ΔE*_IE_, we performed light-bias and voltage dependent EQE measurements with the corresponding normalized EQE response at 550 nm shown in Figure 3a and Figure 3b, respectively. Starting with light-bias dependence of the EQE shown in Figure 3a, 2FTp shows a weak light intensity dependence like PM6, while all other polymers show a stronger light-bias dependence. As for *J*_sc_ and FF (Figure 2a), selenophene substitution has different effects for the different side groups, increasing the light-bias dependence for the Tp devices and decreasing it in the CN devices. The voltage-bias dependence of the EQE shown in Figure 3b shows an inverse correlation with the offset *ΔE*_IE_: the three fluorinated devices (PM6, 2FTp, and 2FSe) with larger offsets display a bias independent quantum efficiency, contrary to the CN devices (CNTp and CNSe) with smaller offsets for which the EQE increases strongly with the applied voltage. A similar voltage dependence is observed for Se- and Tp-based systems with the same functional group. The dependence of the EQE on voltage bias is wavelength independent for all devices (Figures S14–16).

Generally, decreasing photocurrent quantum efficiency with increasing light can be assigned to poor competition of charge-carrier collection with bimolecular charge recombination, which may result from high recombination coefficient or low charge-carrier mobility.^41,42^ Increasing voltage-bias dependence indicates poor charge separation or poor carrier collection. When both the light and the voltage-bias dependence of the EQE are low, as seen in PM6 and 2FTp, it implies that there is little bimolecular recombination and that most charges are already successfully separated and extracted at zero bias. Conversely, when the EQE depends strongly on both light and voltage bias, the device efficiency is impacted by both free charge generation (separation) and poor competition between extraction and recombination processes, as seen in both CN devices. In the case of 2FSe; however, we observe increased light-bias dependence combined with minimal voltage-bias dependence, which suggests that this device has relatively high charge-generation efficiency, but high bimolecular recombination rates. However, light-bias dependent *JV* measurements show that the devices are not limited by recombination at short circuit, as shown in Figure S12. Lastly, transient photocharge (TPQ) measurements^43^ were used to determine the charge carrier lifetime at different light intensities, which revealed a lifetime of ∼300 ns at one sun with little difference between the studied materials (Figure S18). The lifetimes decrease with increasing light intensity in a similar way for all devices.

We further performed EL measurements (absolute values shown in Figure 3c and normalized data in the inset) to gain more insight into the energy and emission properties of the emissive states present in the blends. The spectral shape of emission from all four devices (and PM6) is very similar and is dominated by Y6 singlet exciton emission, as confirmed by its similarity to the EL emission of pristine Y6 (inset Figure 3c) and blend PL emission spectra (Figure 2c). Both the EL spectra and the sensitive EQE bandgap tail measurements (Figure 3d) are very similar across all devices and do not show any additional features that would clearly correspond to a distinct CT state emission. This means that the CT state is not very emissive or is energetically close to the singlet state. However, the absolute EL intensity at fixed injection current varies considerably, with the CN devices showing the strongest EL emission that is more than an order of magnitude higher than that of the corresponding 2F devices. Given a particular functional group, the Tp devices show a somewhat stronger EL than the Se devices. Thus, the strength of the EL emission correlates well with the inverse of *ΔE*_IE_ and the trends observed in *V*_oc_. The same trend in intensity and almost identical spectral shapes is seen in the PL in Figure 2c. Viewed on its own, the comparatively brighter PL intensities in the CN and the Se devices could suggest either poor exciton dissociation efficiency or low nonradiative losses (but higher radiative flux). The former is supported by an increased voltage dependence of the PL in the CN devices (Figure S13). At the same time, we expect to see low nonradiative losses, because of the increased EL intensity despite similar EQE tail shape and optical bandgap. Together, these data indicate that there are indeed both low nonradiative voltage losses alongside reduced exciton dissociation in the devices with smaller *ΔE*_IE_ (CNTp and CNSe).

To confirm this, we calculate the voltage losses from the EQE and EL measurements (Figure 3d) according to a previously published method,^44,45^ with the result shown in Table 2. The radiative limit to *V*_oc_ (obtained from the EQE measurements) is very similar for all four device types, but the measured open circuit voltage is larger in the CN devices. This results in strongly reduced nonradiative voltage losses in the CN devices, with a very low (0.17 V) nonradiative voltage loss obtained for the CNTp-based device, which also has the lowest offset *ΔE*_IE_.

**Table 2:** Open Circuit Voltage (*V*_oc_), Radiative Open Circuit Voltage (*V*_oc,rad_), and the Nonradiative Voltage Losses (Δ*V*_oc,nr_) That Were Calculated from the EL and the EQE Measurements

	*V*_oc_ (V)	*V*_oc,rad_ (V)	Δ*V*_oc,nr_ (V)
**PM6:Y6**	0.83	1.1	0.26
**2FSe:Y6**	0.80	1.1	0.29
**2FTp:Y6**	0.84	1.1	0.25
**CNSe:Y6**	0.90	1.1	0.19
**CNTp:Y6**	0.93	1.1	0.17

## Hole Mobility and Surface Morphology

**Figure 4:**
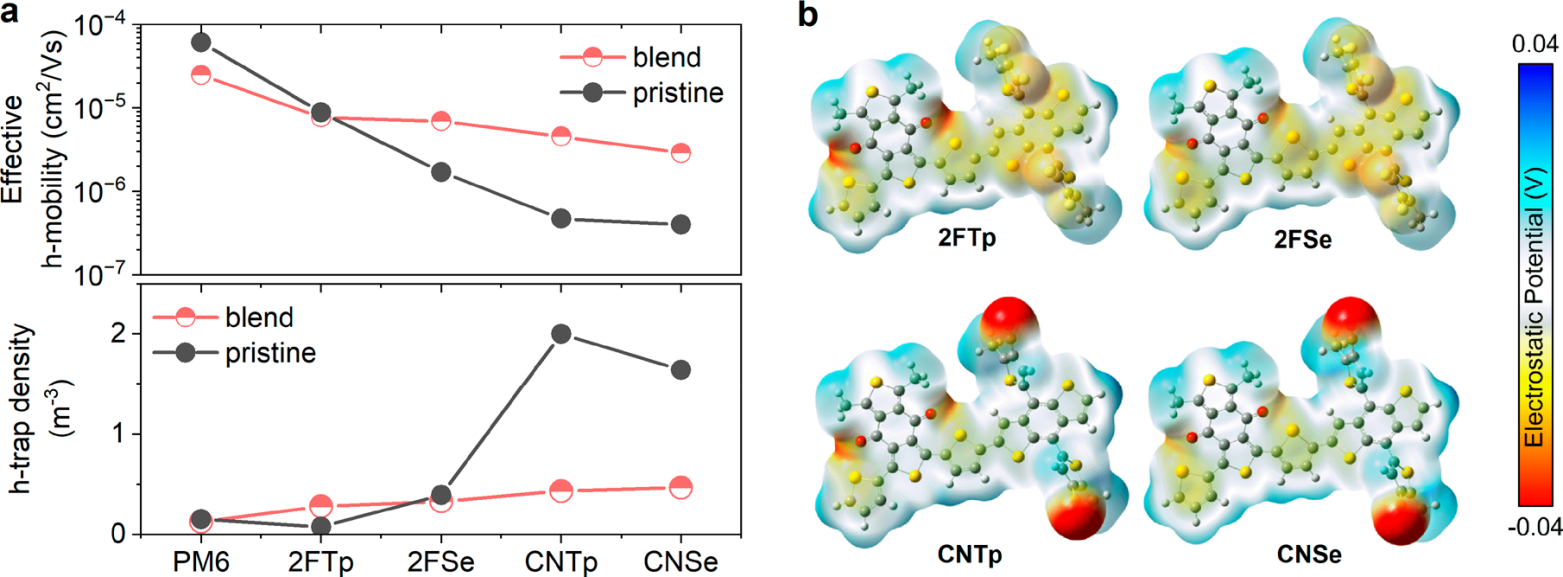
(a) Effective hole mobility at a charge carrier density of 1 × 10^21^ cm^–3^ (considering occupation of trap states) and the total density of localized hole trap states in pristine polymers and polymer:donor blends. Trap free mobilities, the effective density of trap states per unit energy, and the characteristic energy of the exponential tail are shown in Figures S21 and S22 and Table S1. (b) Charge population analysis of the monomer versions of the four PBDB-T derivative polymers. We show the electrostatic potential mapped onto the electron density surface (isosurface) of the molecules, which is a measure of the charge density. The regions with a more negative electrostatic potential (red) correspond to a higher charge density, while the regions with a positive electrostatic potential (blue) correspond to a negative charge density (hole character). The simulations were performed with Gaussian, and the visualization was obtained from GaussView.

In order to establish the possible contribution of charge transport to the different behavior observed, we performed space-charge-limited-current (SCLC) hole mobility measurements on single carrier (hole only) devices. We fit the dark current–voltage curves with the device model “gpvdm”,^46−48^ which accounts for energetic disorder via tails of trap states, and injection barriers (more details in the Supporting Information, Section 3 and Section 10). With the model, we determine the effective hole mobilities and hole trap density in pristine polymer films and in polymer:Y6 blend films as shown in Figure 4a. In the pristine polymer films, we find that the hole mobility is considerably reduced in all four new polymers, in contrast to the PM6 reference. Further, the CN devices show reduced effective hole mobility compared to the 2F devices due to a strongly increased hole trap density, while for either set, substituting thiophene by selenophene causes a further, smaller, reduction in the hole mobility. The blend films show the same trends of a higher hole trap density and reduced mobility in the CN polymer than in 2F-polymer blends; however, these changes are much less pronounced than in the pristine films. The smaller difference could be due to the high ambipolar mobility of Y6^49^ compensating for a part of the lost hole mobility in the blends.

We find a possible explanation for the increased number of tail states in the CN polymers based on the calculated charge density distributions in the different polymers using DFT. As shown in Figure 4b, the CN polymers display a much more polarized charge distribution. It has been shown in previous theoretical studies that a random distribution of permanent dipoles in an amorphous structure increases the local variation in the electrostatic potential, thus creating a more heterogeneous energetic landscape through which the charges move.^50^ In general, an increased density of trap states makes it harder for charge collection to compete with charge recombination.

Lastly, we investigate the morphology as a possible explanation for the differences in mobility using atomic force microscopy (AFM) on pristine films and blends, shown in Figure S19 and Figure S20 respectively. The microstructure of both, pristine and blend films, containing PM6 and CNSe appears more structurally ordered than the other materials. This observation agrees with the stronger broadening in the absorption spectra of CNSe (Figure S3) between solution and film, which often indicates more delocalized states due to more structural order. However, the surface morphological appearance cannot explain the overall trend in the observed mobilities.

## Modeling

Summarizing our results so far, we have presented four new polymers with a range of ionization energies and hence with a range of Δ*E*_IE_ relative to the acceptor, Y6, achieved through either backbone selenophene substitution or difluorination or dicyanation of side groups. We find that difluorination of PBDB-T side groups has minimal to no effect on the IE energy level and mainly negatively affects the mobility. In comparison, cyanation of the same side groups leads to a strong downshift in energy levels, while thiophene to selenophene substitution in the backbone decreases the bandgap due to slightly raised IE energy levels. As expected, reducing Δ*E*_IE_ consistently increases *V*_oc_ and decreases Δ*V*_oc,nr_ across all devices; however, strongly reduced Δ*E*_IE_ is also accompanied by negative effects such as poor charge separation and strong charge-carrier recombination. Interestingly, there are different effects of reducing Δ*E*_IE_ through selenophene substitution on charge-carrier recombination depending on whether the polymer was fluorinated or cyanated. In the former, the recombination is enhanced leading to low *J*_sc_ and FF, while in the latter selenophene substitution improves those properties thanks to reduced recombination.

While the changes in *V*_oc_ and nonradiative voltage losses can be easily explained with the IE energy levels, it is unclear why the photocurrent generation is so drastically compromised in the CN systems even though neither nonradiative recombination at open circuit nor mobility seem to suffer much more than their difluorinated counterparts. We suspect that the lower charge-generation efficiency is caused by the reduced energetic offset between the donor and the acceptor ionization energies Δ*E*_IE_; however, it is unknown if the losses are mainly caused by a poor exciton dissociation rate *k*_LE→CT_^dis^ or poor CT dissociation rate *k*_CT→CS_^dis^ or charge pair reformation.

**Figure 5:**
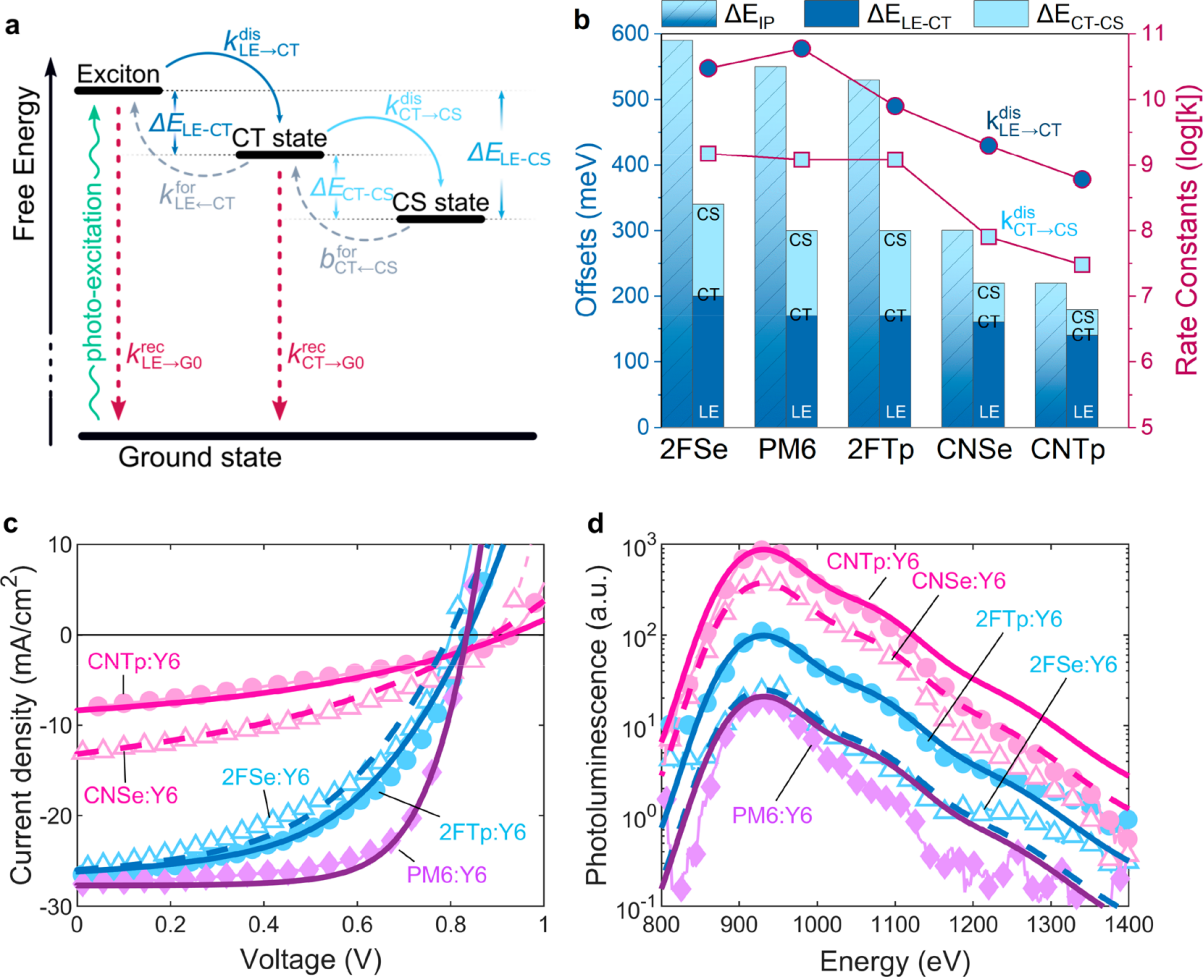
(a) Free energy level diagram of the states and transitions that are considered in the model. (b) Comparison of the offsets Δ*E*_LE–CT_ and Δ*E*_CT–CS_ alongside the dissociation rates *k*_LE→CT_^dis^ and *k*_CT→CS_^dis^ that were obtained from the modeling for all five materials. Materials are sorted by their experimentally determined Δ*E*_IE_, decreasing from left to right. (c,d) Simultaneous fitting of the the *JV* scan (c) and the photoluminescence (d) using the computational model. The data were fitted using the Δ*E*_LE–CT_, the *k*_LE→CT_^dis^, the *k*_CT→CS_^dis^, and μ_eff_^h^ as free parameters.

To answer this question, we use a recently demonstrated numerical model developed by Azzouzi et al.,^51^ which combines a one-dimensional semiconductor device model called DriftFusion^52^ to describe the charge carrier transport and a semiclassical rate model which provides the rate constants (dissociation and recombination) for the processes involved in pair generation and recombination in the active layer.^51^ The three-state model, incorporating a local exciton (LE) state, a charge-transfer (CT) state, and a charge-separated (CS) state, with a kinetic representation of the considered generation and recombination processes between the states, is shown in Figure 5a. The rate model uses Marcus–Levich–Jortner to calculate the (non)radiative recombination rates of excited states, from which the population density of [LE]_0_, [CT]_0_, and [CS]_0_ states at equilibrium are determined via the continuity equations. The energy of the CS state (difference between IE and electron affinity) is related to the other parameters via
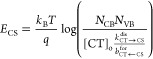
where *N*_CB_ and *N*_VB_ are the effective density of states in the conduction and valence bands, respectively, and *b*_CT←CS_^for^ is the CS to CT reformation rate constant.^51^

We used the model to simultaneously fit the experimental *JV* and PL data. Here, the *JV* curve provides us with information about the offset Δ*E*_LE–CT_ between the exciton and CT state (through the *V*_oc_), the charge-generation efficiency (through *J*_sc_), and the mobility (through the FF). The PL quenching gives us information about the exciton dissociation rate (by considering the absolute emission strength). To reproduce the experimental results for the blends in the series, we consider four free parameters changing along the series: 1) we tune the energetic offset Δ*E*_LE–CT_ to reproduce the trend in the open circuit voltage; 2) and 3) we adapt the LE and CT dissociation rates (*k*_LE→CT_^dis^ and *k*_CT→CS_^dis^, respectively) to fit the short circuit current as well as the photoluminescence emission strength; and 4) we use the hole mobility μ_eff_^h^ to account for the different FFs. Relating to no. 4, we use the SCLC derived hole mobility values for comparison with the trends inferred from fitting data, and not as reliable quantities that have to be reproduced. We note that varying all four parameters was necessary to achieve a good agreement with the experimental data. The impact of each of these parameters on simulated data is further explained in the Supporting Information (Section 11, Figure S23, Table S2, Table S3). All other parameters were kept constant between the different polymers once a suitable set of values was found. Aside from the simulated *JV* and PL, the model provides information about the offset between the CT and the CS state Δ*E*_CT–CS_ as output.

The best fitting input parameters and the resulting output parameters, in comparison with the experimental values, are shown in Table 3 and summarized in Figure 5b. The resulting fits of the experimental *JV* and PL data are shown in Figure 5c,d, respectively. According to these fits, we find that lower voltage losses and an increased *V*_oc_ in the device correlate well with a low offset Δ*E*_LE–CT_. Fitting the PL measurements suggests that the reduction of this energetic offset leads to a lower exciton dissociation rate, *k*_LE→CT_^dis^. However, we find that the exciton dissociation rate alone cannot explain the low *J*_sc_ values in the CN devices. Indeed, in those devices, the CT dissociation rate constant *k*_CT→CS_^dis^ is reduced by over an order of magnitude in addition to the lower *k*_LE→CT_^dis^. In accordance with this, the modeling reveals that Δ*E*_CT–CS_ is most strongly reduced in the CN devices. These findings explain both the anticorrelated behavior of the PL and the EQE that was shown in Figure 2b,c (indicating lower exciton dissociation) as well as the strong voltage-bias dependence that was observed only in the CN devices as shown in Figure 3b (suggesting poor charge separation). In our system it appears that for large enough offsets a reduction in Δ*E*_IE_ has only a minor impact on Δ*E*_CT–CS_ but that beyond a certain threshold (very low Δ*E*_IE_) the Δ*E*_CT–CS_ is much more strongly reduced than Δ*E*_LE–CT_. It might be the case that this happens once the CT state starts to hybridize with the excitonic state in line with previous findings by Eisner et al.^53^ Thus, according to our model, when Δ*E*_CT–CS_ is reduced much more strongly in low-offset systems, it acts as a limiting factor for the charge-generation efficiency. This would be consistent with the observation that while selenophene substitution increases the charge generation in the CN devices (where *k*_CT→CS_^dis^ is limiting), it has little impact in the 2F devices (where *k*_CT→CS_^dis^ remains constant). Our findings suggest that the efficiency of low offset systems could potentially be improved if we can decrease Δ*E*_IE_ without disproportionately reducing Δ*E*_CT–CS_ such that the total energy difference between exciton and free charge Δ*E*_LE–CS_ is distributed more equally between Δ*E*_LE–CT_ and Δ*E*_CT–CS_.

**Table 3:** Summary of Input Parameters That Produce the Best Fit between Experimental Data and Simulation, as Shown in Figure 5 alongside Resulting Device Parameters Obtained from the Simulation and the Comparison to the Experimental Device Data^a^

	Input Parameters				Simulation Output					Experiment					
	Δ*E*_LE–CT_ (eV)	*k*_LE**→CT**_^dis^ (cm^3^ s^–1^)	*k*_CT**→CS**_^dis^ (cm^3^ s^–1^)	μ_eff_^h^ (cm^2^ s^–1^ V^–1^)	Δ*E*_CT–CS_ (eV)	*J*_SC_ (mA/cm^2^)	FF (%)	*V*_OC_ (V)	Δ*V*_nr_ (mV)	Δ*E*_IP_ (eV)	*J*_SC_ (mA/cm^2^)	FF (%)	*V*_OC_ (V)	Δ*V*_nr_ (V)	μ_eff_^h^ (cm^2^ s^–1^ V^–1^)
**2FSe:Y6**	0.20	3e10	1.5e9	9e-5	0.14	26.0	48	0.80	0.24	0.59	26.1	47	0.80	0.29	7.0e-6
**PM6:Y6**	0.17	6e10	1.2e9	5e-4	0.13	27.2	68	0.83	0.21	0.55	27.4	68	0.83	0.26	2.5e-5
**2FTp:Y6**	0.17	8e9	1.2e9	9e-5	0.13	26.1	50	0.83	0.21	0.53	26.5	51	0.84	0.25	7.7e-6
**CNSe:Y6**	0.16	2e9	8e7	5e-5	0.06	13.2	36	0.89	0.15	0.30	13.3	40	0.90	0.19	4.6e-6
**CNTp:Y6**	0.14	6e8	3e7	5e-5	0.04	8.4	37	0.92	0.13	0.22	8.3	34	0.93	0.17	2.9e-6

a The devices are sorted with decreasing offsets of Δ*E*_LE–CT_.

In summary,in this work we explored the processes limiting charge generation in low-offset systems by studying four newly synthesized PBDB-T derivative donor polymers, in addition to PM6, and the resulting organic bulk-heterojunction solar cells that were fabricated with Y6 as the acceptor. We identify the higher hole mobility in PM6 as the main reason for why it outperforms the novel donor polymers, which appear to suffer from “over”-fluorination.^54^ In line with our expectations, we find that the more electron withdrawing nitrile side groups reduce the ionization energy, while selenophene substitution in the backbone bridge decreases the bandgap. This leads to a gradual tuning of the energetic offset between exciton and CS state, which we use to investigate the charge-generation properties. We find that the offset between the donor and acceptor ionization energies in the cyanated devices (<0.30 eV) is too small for efficient charge generation, while larger offsets (>0.53 eV) provide close-to unity charge generation. Interestingly, we find that CT-CS offset is reduced disproportionately compared to the LE-CT offset. While the reduced ionization energy offset impairs both exciton as well as CT dissociation rates, we identify the latter as the main factor responsible for the low charge generation in the CN devices. Our findings suggest that the efficiency in low-offset systems could be improved if the offset between donor and acceptor ionization energies can be reduced without (or only minor) impact to the CT dissociation rate. In such a system a high open circuit voltage thanks to low nonradiative voltage losses could potentially be achieved with only minor losses in charge-generation efficiency.

## Supporting Information Available

1. Material Synthesis, 2. Device Fabrication, 3. Characterization Methods, 4. Material Analysis, 5. Device Current–Voltage Characteristics, 6. Bias Dependent Photoluminescence 7. EQE Voltage Dependence at different Wavelengths 8. Charge Carrier Lifetimes, 9. Surface Morphology, 10. SCLC fitting with gpvdm, 11. Numerical Modeling (PDF)

## Notes

^#^ J.S.M. email: j.muller21@imperial.ac.uk.
