# Supplementary material for: Charge-Transfer State Dissociation Efficiency Can Limit Free Charge Generation in Low-Offset Organic Solar Cells
Source: ACS Energy Lett. 2023 Jul 14;8(8):3387–97. doi: 10.1021/acsenergylett.3c00943 (PMC10425975; doi:10.1021/acsenergylett.3c00943)
Supplement: Supplementary file 1 — nz3c00943_si_001.pdf [file nz3c00943_si_001.pdf]

Charge-transfer state dissociation efficiency can limit free charge generation in low-offset organic solar cells

Jolanda Simone Müller,[+ a] *Marc Comí,*[+ b] *Flurin Eisner,** [a] *Mohammed Azzouzi,* [a] *Diego Herrera Ruiz,* [a] *Jun Yan,* [a c] *Salahuddin Sayedshabbir Attar,* [b] *Mohammed Al-Hashimi,** [b] *and Jenny Nelson** [a]

[a] Department of Physics and Centre for processable Electronics, Imperial College London, Blackett Laboratory, Prince Consort Road, London SW7 2AZ, United Kingdom

[b] Department of Arts and Sciences, Texas A&M University at Qatar, Education City, P. O. Box 23874, Doha, Qatar

[c] School of Science and Engineering, The Chinese University of Hong Kong, Shenzhen, Guangdong Province 518172, P. R. China

[+] joint first; *Corresponding authors

Supplementary Information

1. Material Synthesis

The synthesis of monomers **M1** and **M2** is depicted in Figure S1 [1,2]. The Stille cross-coupling reaction of 1,3-dibromo-5,7-bis(2-ethylhexyl)-4H,8H-benzo[1,2-c:4,5-c']dithiophene-4,8-dione (BDTD) with tributyl(selenophen-2-yl)stannane and trimethyl(thiophen-2-yl)stannane in chlorobenzene (CB) using $Pd(PPh_3)_4$ as the catalyst afforded intermediates **1** and **2** in a good yield. Subsequently, dibromination with N-bromosuccinimide (NBS) in dimethylformamide (DMF) afforded the desired target monomers **M1** and **M2**.

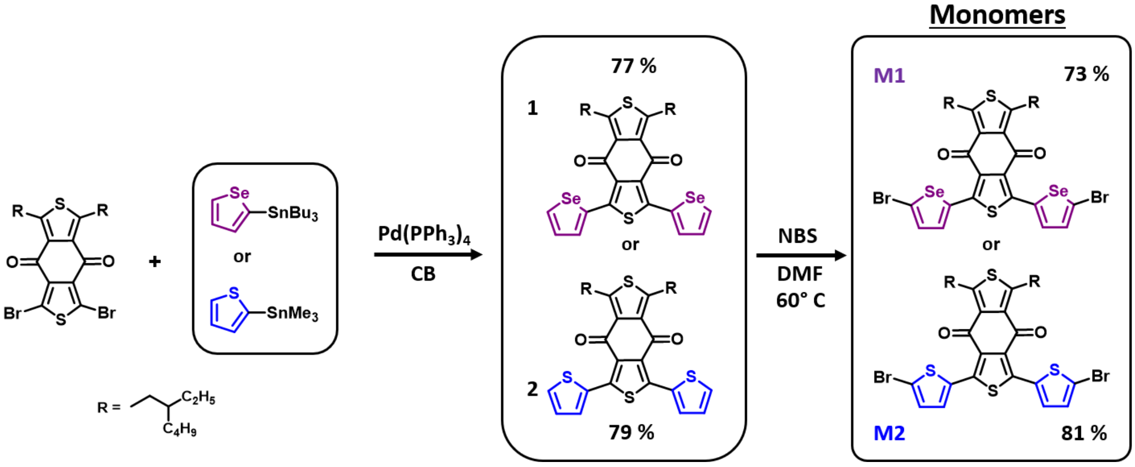

Figure S1: *Synthesis of monomers M1 and M2 (R = 2-ethylhexyl).*

As depicted in Figure S2 copolymers **2FSe**, **2FTp**, **CNSe** and **CNTp** were synthesized using Stille cross-coupling polymerization of BDTD monomers (**M1** and **M2)** with the respective benzodithiophene (BDT) monomers (**2F** and **CN**) in a microwave reactor in chlorobenzene using $Pd(PPh_3)_4$ as the catalyst. All copolymers were precipitated in acidified methanol and purified via Soxhlet extraction with a sequence of refluxing methanol, acetone, hexane and chloroform. Copolymers **2FSe**, **2FTp**, **CNSe** and **CNTp** were collected in chlorobenzene solution and isolated as blue dark solids. Number average molecular weights (Mn) and polydispersities (Đ) of the copolymers were measured, resulting in high molecular weights in the range of Mn= 90-130 KDa with Đ of 2.3-2.4.

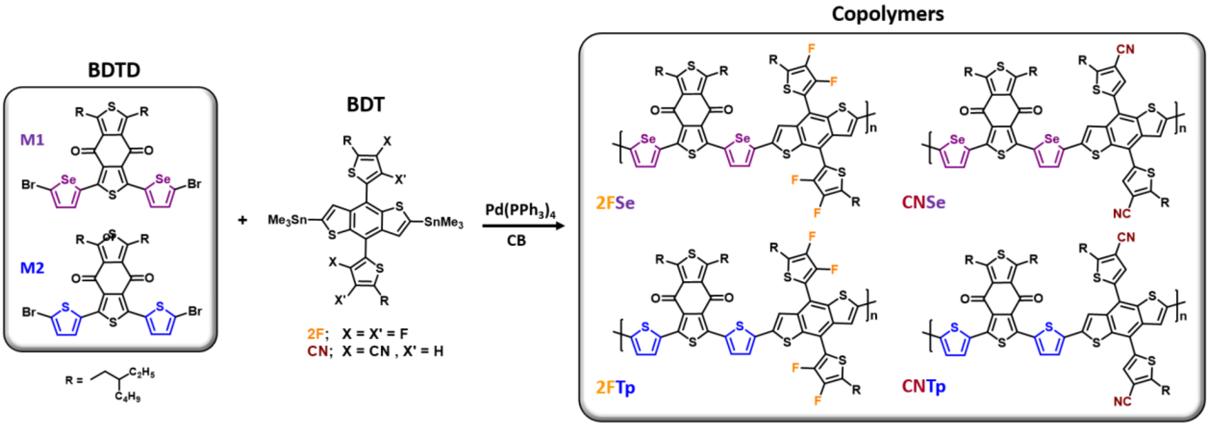

Figure S2: Synthesis of 2FSe, 2FTp, CNSe and CNTp.

All commercially available solvents, reagents, and chemicals were used as received without further purification unless otherwise stated. All operations and reactions were conducted under argon using standard Schlenk line techniques. Microwave experiments were performed in a Biotage initiator V 2.3.

2. Device Fabrication

Solar cell devices were fabricated with the structure: ITO/PEDOT:PSS/Donor:Acceptor(1:1.2)/PFN-Br/Ag on 2 cm x 2 cm ITO coated glass substrates, purchased from XXX. The substrates were washed with sonication in water with decon90, deionized water, and 2-propanol for 15 min each. Afterward, the samples are dried with a nitrogen gun and treated with UV-Ozone for 20 min. PEDOT:PSS Al 4083 was filtered (0.45 µm), 150 µl were spin-coated (static spin-coating with an SPS SPIN150 at 5000 rpm for 60 s and annealed at 150 °C for 15 min). Solutions for the active layer were prepared at 1:1.2 donor:acceptor ratios in chloroform with the following concentrations: 9 mg/ml 2FTp:Y6, 12 mg/ml for 2FSe, 11 mg/ml for CNTp, 12 mg/ml for CNSe, each with 0.5% chloronaphthalene. Solutions were stirred for 2h at 30 °C. Then, 30 µl were spin-coated (dynamic spin-coating) at 2000 rpm for 30s inside a nitrogen atmosphere (glovebox). The electron transport layer (5 nm PFN-Br) was spin-coated (dynamic spin-coating) using 50 µl of 0.5 mg/ml PFN-Br in Methanol, at 2000 rpm for 40 s. The active layer was annealed at 100 °C for 8-10 min on a Wenesco hot plate. The top contact electrodes (80 nm Ag) were evaporated using a mbraun MB 200B evaporator with a deposition rate of 0.3 A/s, under vacuum with a pressure below 10^{-6} bar. The fabrication of the hole transport SCLC devices was identical, apart from replacing the PFN-Br layer with 7 nm MoO_3 evaporated in the same way as the Ag electrodes. Thus, leading to the structure: ITO/PEDOT:PSS/Donor:Acceptor(1:1.2)/MoO_3/Ag.

3. Characterisation Methods

Thickness: Film thicknesses were measured with a Bruker DektakXT, by scanning across a scratch made with a thin needle. The thickness of PEDOT:PSS and PFN-Br was determined on separate samples and subtracted from the total.

Current density -Voltage (JV): J-V measurements (dark and AM1.5) were performed using a Keithley 236 source measure unit. We used a Newport solar simulator (Ozone free Xenon Arc Lamp, 300 W) to provide the one-sun (AM 1.5) illumination spectrum at an intensity of 100 mW/cm^2. A switchbox was used to measure all 8 pixels in sequence (one pixel = active device area of 5 mm^2). This setup was also used to measure the current-voltage characteristics for the SCLC mobility analysis.

External Quantum Efficiency (EQE): EQE measurements, were performed with either a Stanford Research System SR380 or a Zurich Instruments HF2LI lock-in amplifier (for EQE spectra and voltage and light bias dependant EQE respectively) together with a chopper at 300 Hz. A monochromator (Newport, CS260-RG-4-MT-D) was used to generate the incident monochromatic light beam from a tungsten halogen light source (Newport 6692, 50-500 W). Scattered light from the monochromator was removed with long pass filters at 610 nm, 780 nm, 850 nm and 1000 nm.

Photoluminescence (PL): For the photoexcitation, we use a 473 nm diode laser (Lambda Photometrics, DPBL 9010F with a PHotop LDC-25005 power supply). The resulting radiation emitted by the sample was recorded with a Shamrock 303 spectrograph combined with an iDUS InGaAs array detector (Andor SR 303i-B) cooled to -90 °C. In addition, we put a 595 nm long pass filter between the sample and the detector to remove any scattered laser light. The recorded spectra were normalised to the detector sensitivity, which was determined through calibration with a Halogen lamp. Additionally, a dark measurement for the same integration time was subtracted from each dataset.

Electroluminescence (EL): We used a Keithley 2450 source meter to control the input current in the EL measurements. The EL spectra were recorded with the same spectrometer that was used for the PL measurements (Shamrock 303 spectrograph, iDUS InGaAs array detector (Andor SR 303i-B) cooled to -90 °C) and normalised the same way.

Non-Radiative Voltage Loss: Non-Radiative Voltage Losses were determined according to the method suggested by J. Yao et al. [3], which extends the EQE spectrum with the EL emission according to the reciprocity relation [4] in order to gain a high precision of the radiative limit of the dark saturation current $J_{0,rad} = q \int \phi_{BB} * EQE_{dev}(\lambda)\, d\lambda$. This value is then used to determine the radiative open circuit voltage:

$$V_{oc,rad} = \frac{k_B T}{q} \ln(\frac{J_{sc}}{J_{0,rad}} + 1)$$

With the Boltzmann constant k_B, the elementary charge q, and the temperature T, which is assumed to be room temperature (300 K), and the short circuit current J_{sc}. $V_{oc,rad}$ is then used to determine the non-radiative voltage losses $\Delta V_{oc,nr} = V_{oc,rad} - V_{oc}$.

Ambient Pressure Photoemission Spectroscopy (APS): To determine the LUMO levels, we used KP Technology Ltd's APS02 system, in which the material is illuminated with a tuneable UV-lamp and a gold tip placed close to the surface collects the emitted electrons (photo-electron detector). The measurement is performed under air (ambient pressure).

Gel Permeation Chromatography (GPC): Number-average (M_n) and weight average (M_w) were determined by Agilent Technologies 1200 series GPC running in chlorobenzene at 80 °C, using two PL mixed B columns in series, and calibrated against narrow polydispersity polystyrene standards.

Ultraviolet-Visible-Near-Infrared Absorption Measurements (UV-vis): UV-vis spectra were recorded from 1000 nm to 350 nm on a UV- 1601 Shimadzu UV-vis spectrometer for samples in 10^{-4} M chlorobenzene solution and drop-cast thin film on glass substrate.

Cyclic Voltammetry Analysis (CV) of polymers films were performed under argon atmosphere using a CHI760E Voltammetry analyser with 0.1 M tetra-n-butylammonium hexafluorophosphate in acetonitrile as the supporting electrolyte. A platinum disk working electrode, a platinum wire counter electrode, and a silver wire reference electrode were employed, and the ferrocene/ferrocenium (Fc/Fc+) was used as the internal reference for all measurements. The scanning rate was 100 mV/s. Polymer films were drop-casted from chlorobenzene solutions on a Pt working electrode (2 mm in diameter).

Atomic Force Microscopy (AFM): For our analysis of the surface morphology, we used a tapping mode AFM system (Agilent 5500AFM) in combination with the PicoView control software. To reduce artefact in the AFM images, we a slow scan rate of 1 µm/s. We used Gwyddion for the image analysis, performing the following steps to treat each of the images: “Align Rows”, “Polynomial Background Subtraction”, “Correct Horizontal Scars”, and “Set Zero”.

General-purpose Photo Voltaic Device Model (gpvdm):

Effective hole mobilities and hole trap densities of pristine polymer and blend films are obtained from SCLC current-voltage measurements fitted with the General-purpose Photo Voltaic Device Model (gpvdm), a drift-diffusion model developed by R. Mackenzie [5–7]. For each material, devices of different thicknesses (measured with DektakXT) are fitted simultaneously, as shown in Figure S21 and Figure S22, to obtain the free fitting parameters: the trap-free hole mobility μ_0 ($cm^2V^{-1}s^{-1}$), characteristic energy of the exponential tail E_t (eV), and the effective density of trap states per unit energy N_h ($m^{-3}eV^{-1}$) (In gpvdm called *hole mobility*, h*ole tail slope*, and *hole trap density*, respectively). We assumed the following properties to be constant across all materials: number of free charge carrier density n_f= 1e21, effective density of free hole states N_c = 1e26 m^{-3}, number of traps = 10 bands, gap energy E_g = 1.45 eV, and the room temperature energy k_bT = 25.9 meV. When necessary, the fermi-offset at the contact and parasitic components were adjusted separately. We further assume an exponential density of tail states extending into the band gap in combination with Maxwell-Boltzmann (analytic) free carrier statistics. According to [8] we thus find the total density of localized trap states $N_{\mathrm{tot}}^{h} = N_h \times E_t$ and the effective hole mobility $\mu_{\mathrm{eff}}^{h} = \mu_0 \times \frac{n_f}{(n_f + n_t)}$, where the density of trapped holes $n_t = N_{\mathrm{tot}}^{h} \times (n_f/N_c)^{k_bT/E_t}$, as shown in Figure 4a of the main manuscript. The fitting parameters and calculated n_t and μ_{eff}^{h} are summarised in Table S1.

Time Dependent Density Functional Theory (TD-DFT): We performed TD-DFT calculations with Gaussian 16 [9] version g16-b01-avx2 run on the Imperial HPC supercomputer and used GaussView 6 [10] for additional analyses. We used DFT calculations to determine the optimised geometry of the molecules and subsequent TD-DFT to calculate the excited states and the oscillator strengths. We used the b3lyp functional method with the b3lyp/6-311+g(d,p) basis set for the geometry optimisation and time dependant calculations.

4. Material Analysis

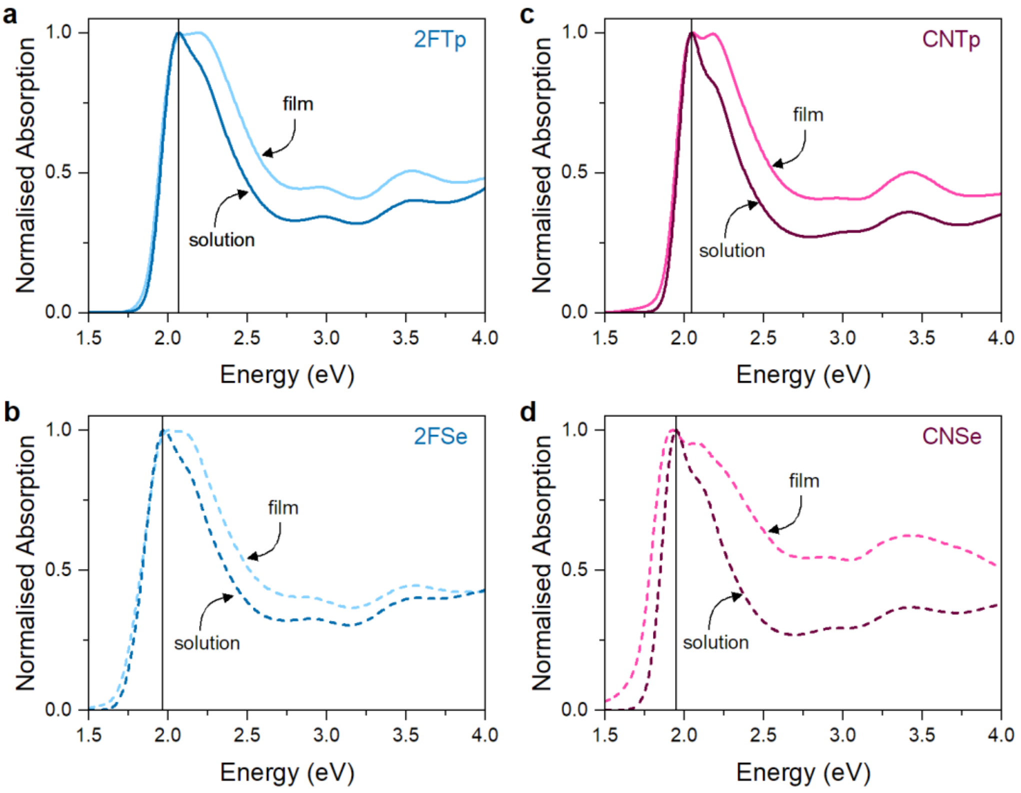

Figure S3: Donor polymer absorption spectra comparing the absorption in solution and in film on glass substrate.

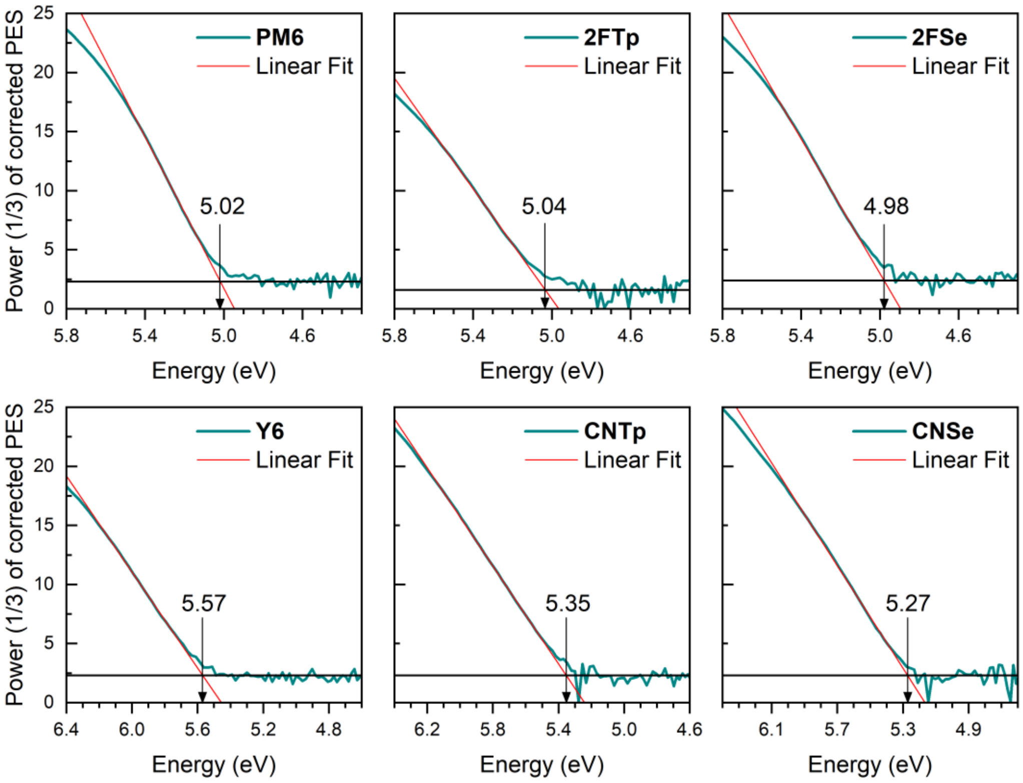

Figure S4: Photoemission Spectroscopy (PES) data acquired with APS. Ionisation potentials are obtained from the intersection between the noise floor and the linear fit of the PES emission (corrected and to the power of 1/3).

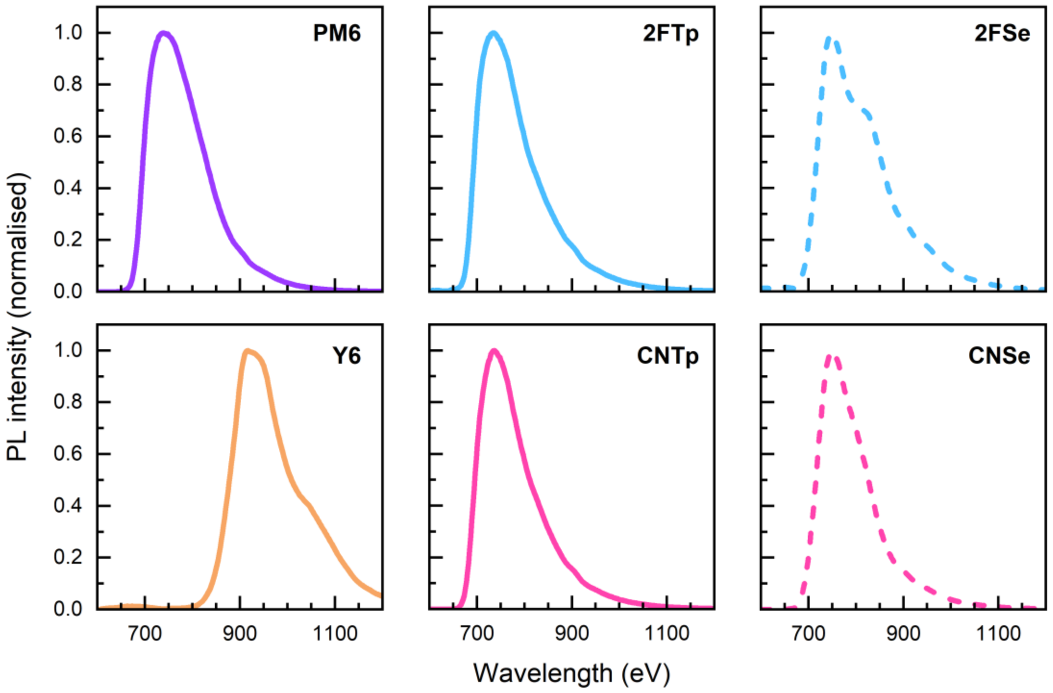

Figure S5: PL spectra of the pristine materials Y6, PM6, 2FTp, 2FSe, CNTp, and CNSe.

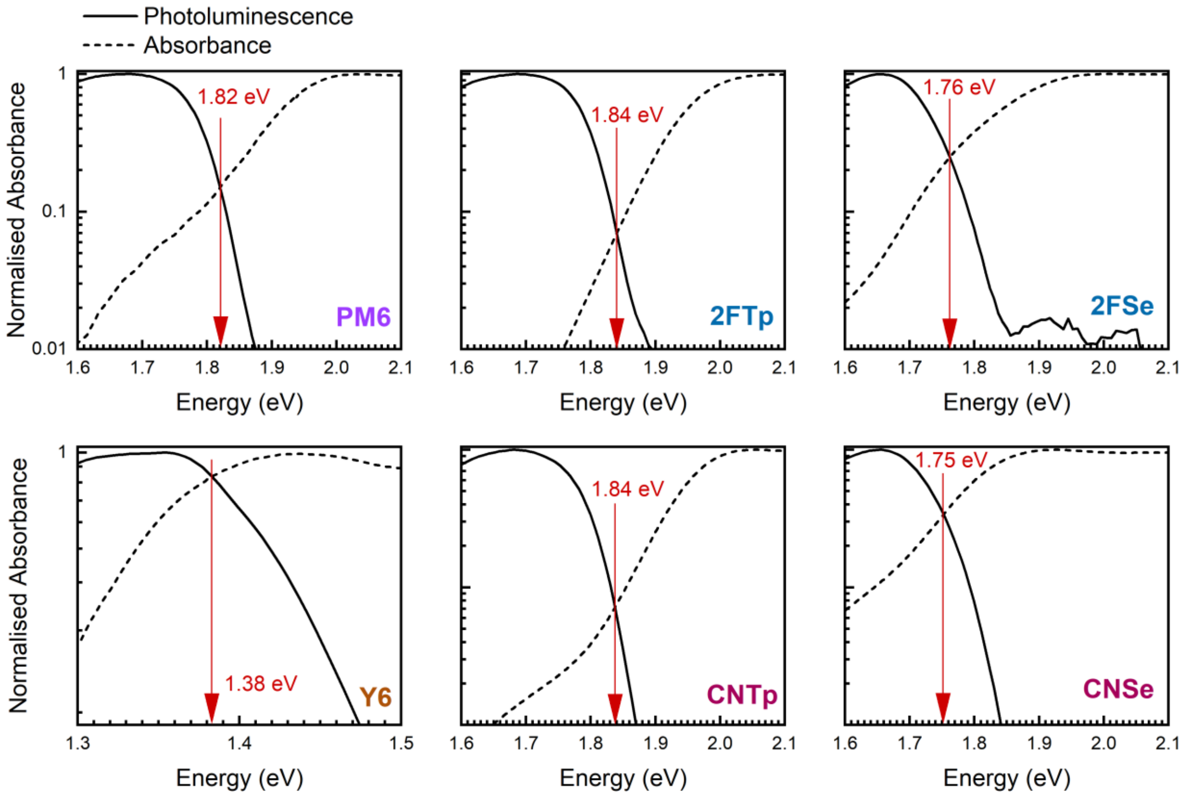

Figure S6: Material bandgaps determined from the intersection between the absorbance and the PL emission. (According to T. Kirchartz et al. in Adv. Energy Mater. (2018))

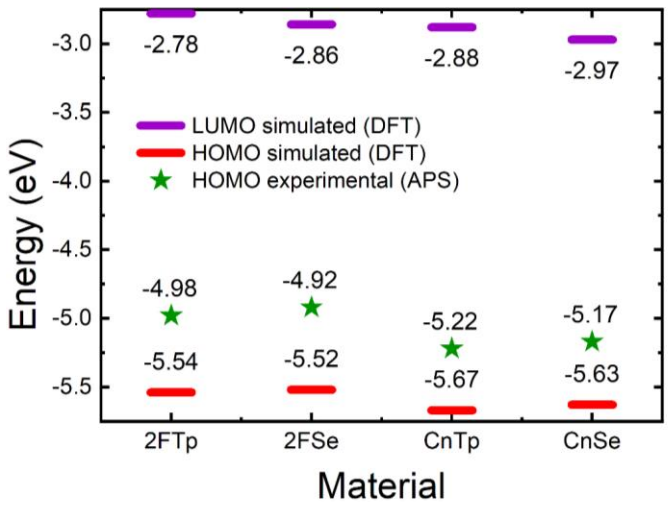

Figure S7: Computationally calculated energy levels compared with the experimentally measured HOMO levels of the four donor polymers

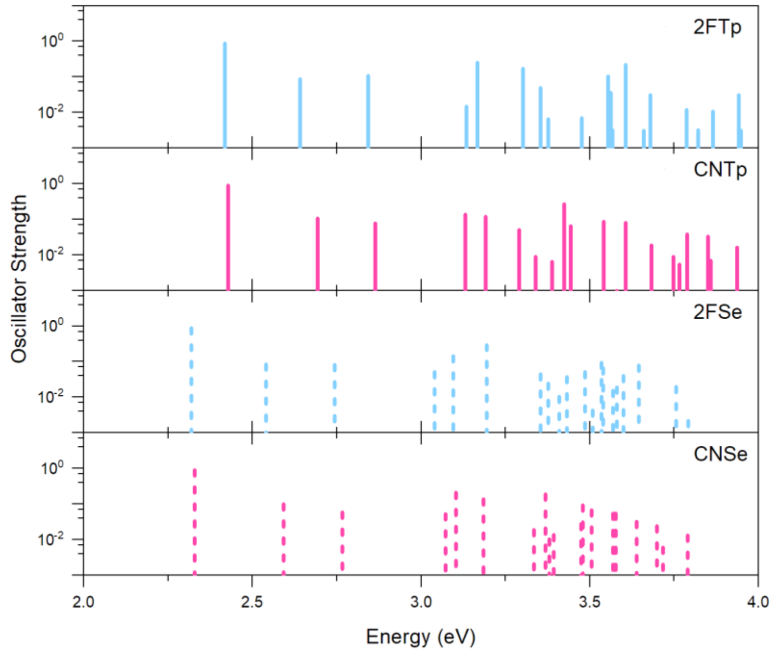

Figure S8 : The oscillator strengths of the energetic transition simulated with TDDFT calculations performed with Gaussian. The calculations were performed on monomer versions of the four donor polymer variants. The transition energies show a red strong redshift upon selenophene substitution, which agrees with the experimentally observed smaller bandgap in the selenophene materials. The nitrile functional group has only a very minor impact on the transition energy.

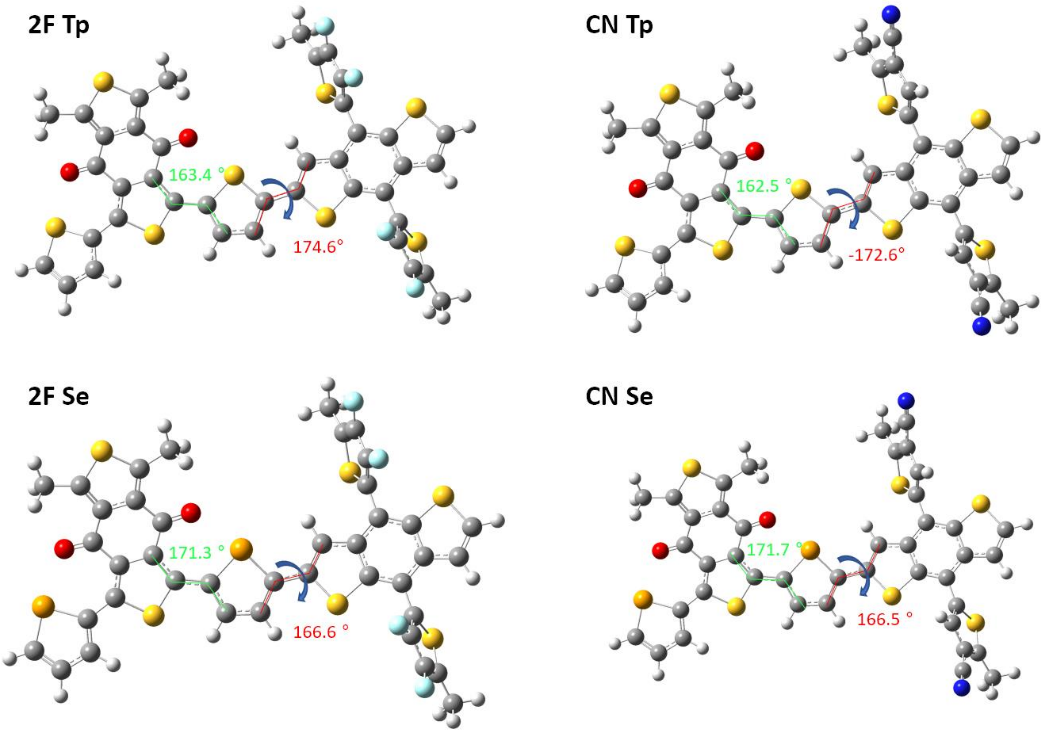

Figure S9: Dihedral angles in the four monomers obtained from DFT calculations that were performed with Gaussian. Thiophene to selenophene substitution in the backbone flattens the dihedral angle connecting the selenophene and the electron acceptor component from 163° → 171° (completely flat would be 180°). Meanwhile the dihedral angle between the selenophene ring and the donor component is increased from 173° → 166°. The change in functional group has only a negligible effect on the dihedral angle.

5. Device Current-Voltage Characteristics

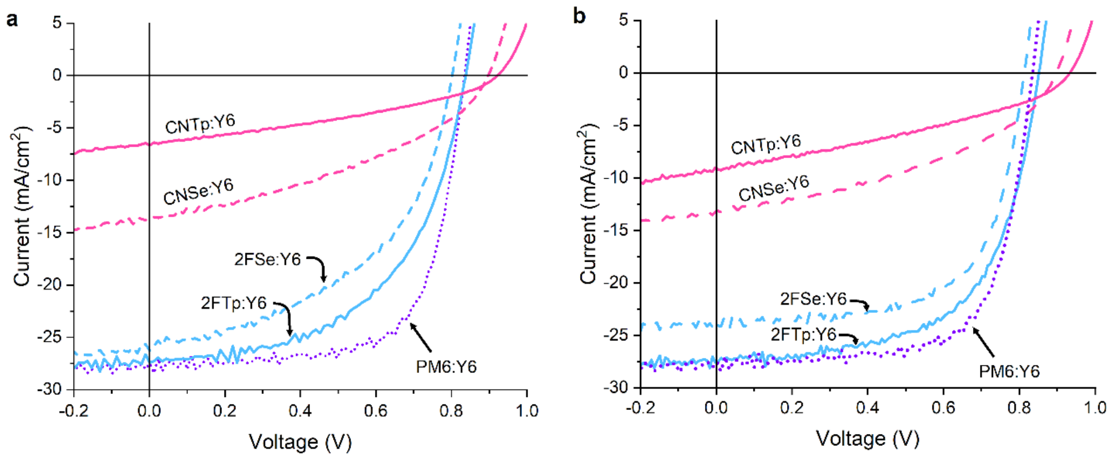

Figure S10: JV characteristics of same thickness devices (a) and best performing devices (b).

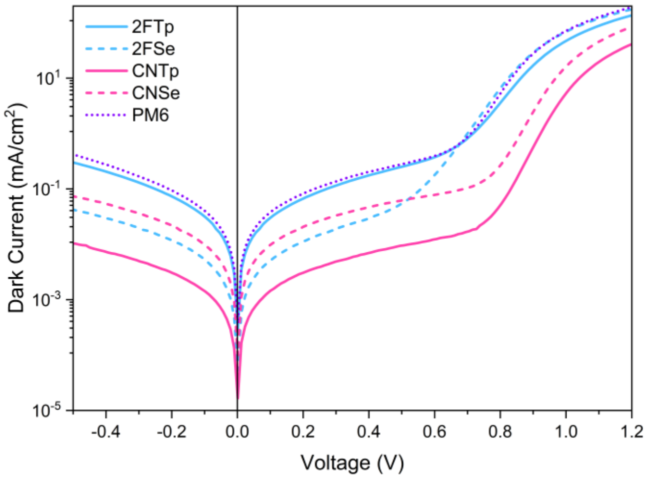

Figure S11: Dark currents of the four same-thickness devices.

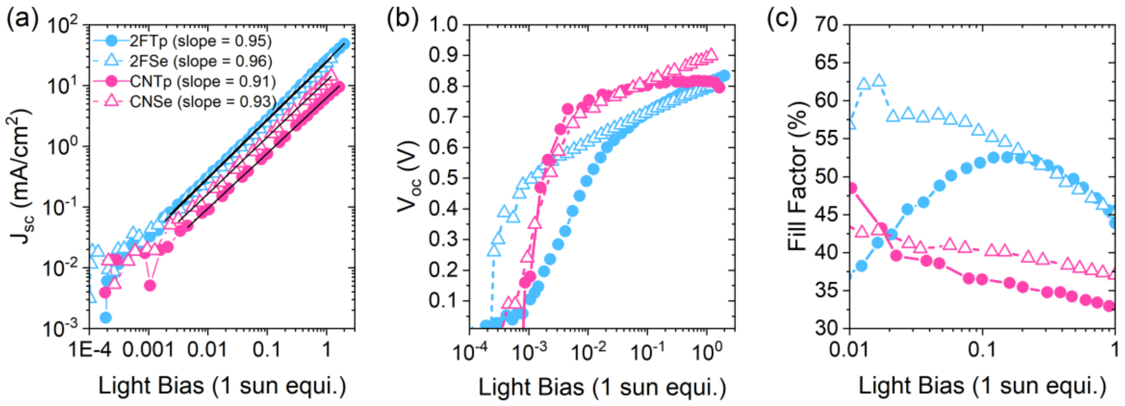

Figure S12: Light bias dependent JV measurements. (a) Light bias dependant J_{sc}. Fitting reveals slopes close to 1 in the log-log plot. (b) Light bias dependent V_{oc} (c) Light bias dependent fill factor.

6. Bias dependent Photoluminescence

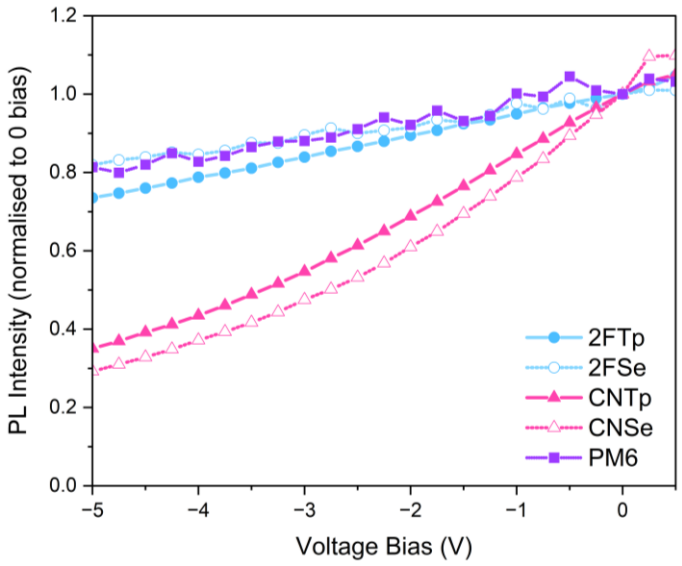

Figure S13: Voltage bias dependent PL peak intensity reduction at 930 nm, normalised to the intensity at 0 bias, illuminated at 473 nm.

7. EQE Voltage Dependence at different Wavelengths and Fluence

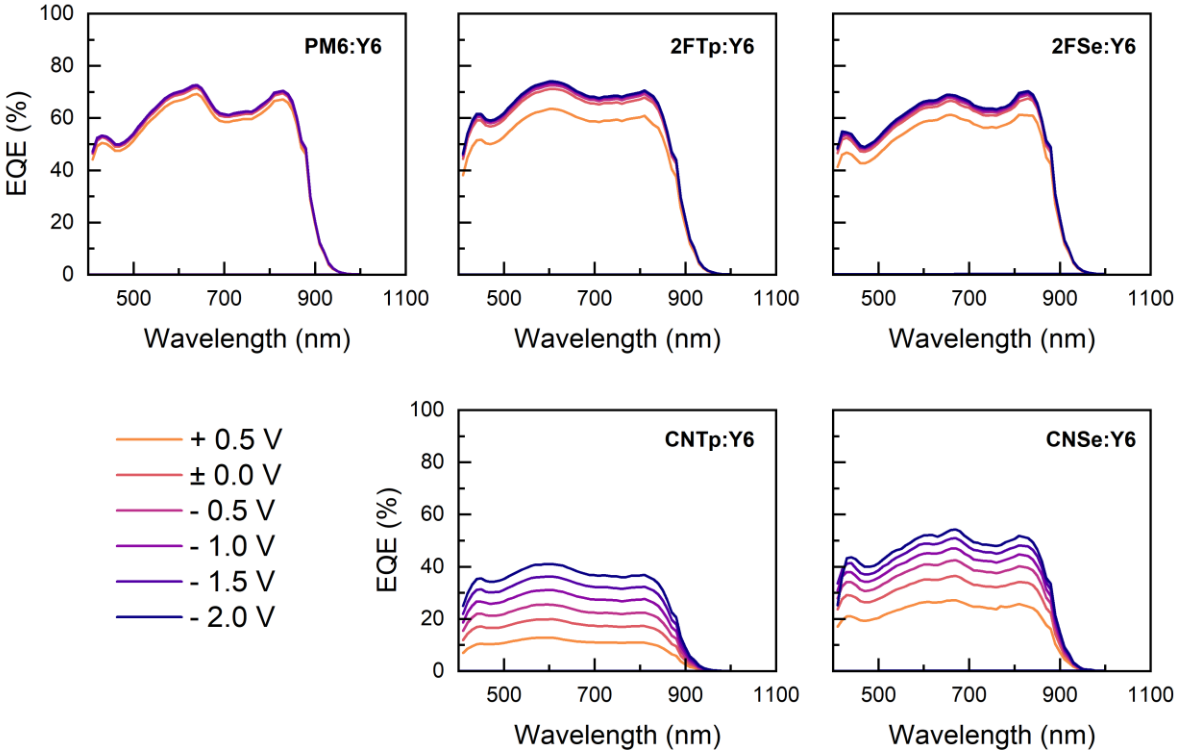

Figure S14: EQE spectra at different applied voltage biases. This measurement was performed on an independent fresh set of devices, which all share a similar thickness.

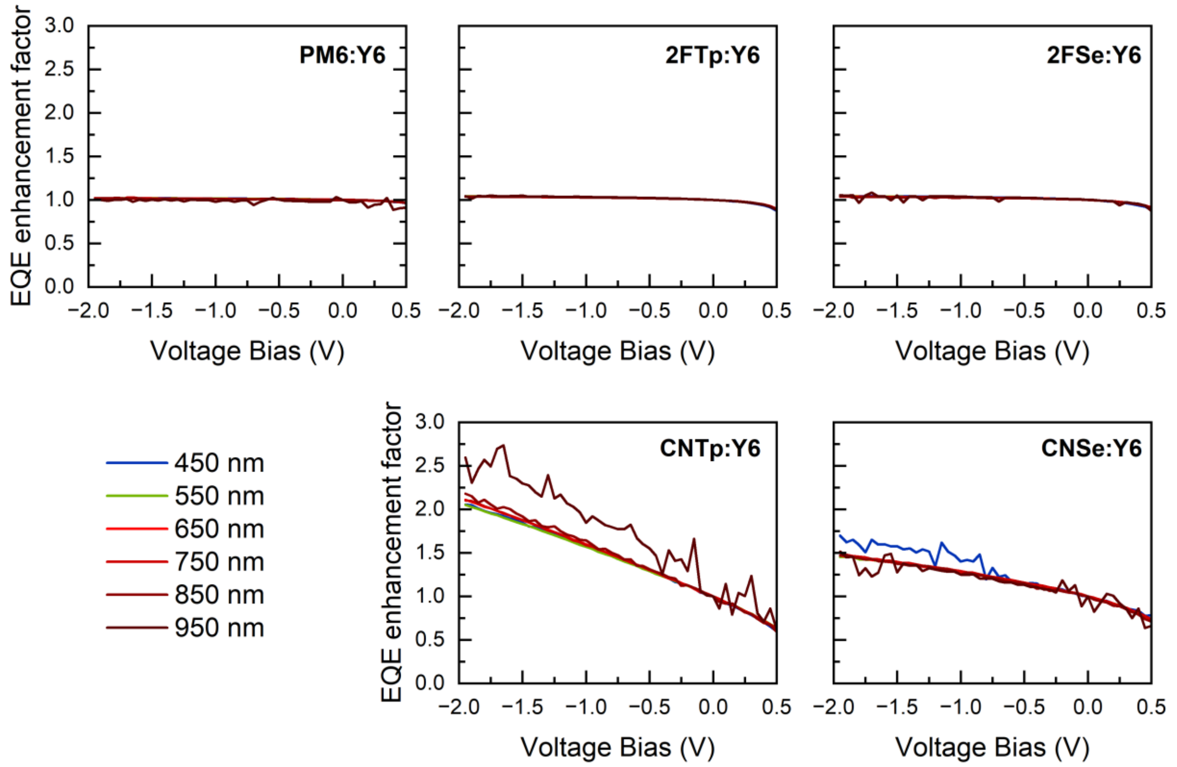

Figure S15: Voltage bias dependent EQE enhancement normalised to 0 V bias at different wavelengths. Not all are visible because they are exactly on top of each other.

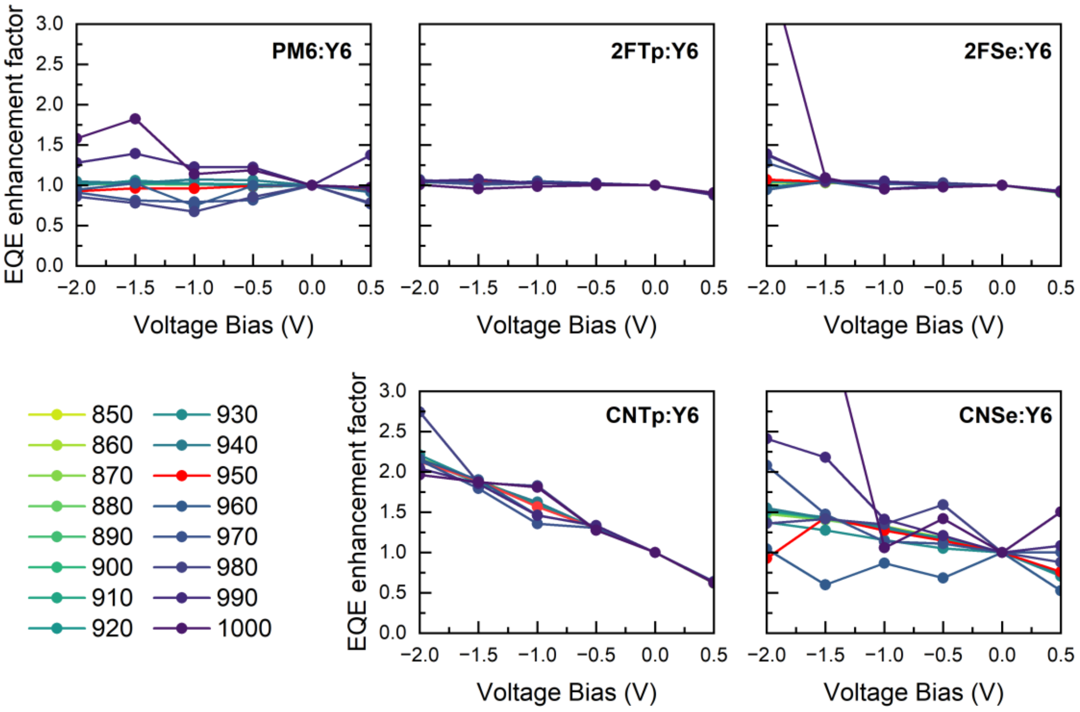

Figure S 16: Voltage bias dependent EQE enhancement normalised to 0 V bias at different wavelengths in the sub-gap region. Data extracted from the wavelength-sweeps in Figure S14. Data at 950 nm is highlighted in red for comparison with the bias voltage sweep in Figure S15. At very low wavelengths the data shows more noise due to very low EQE signals.

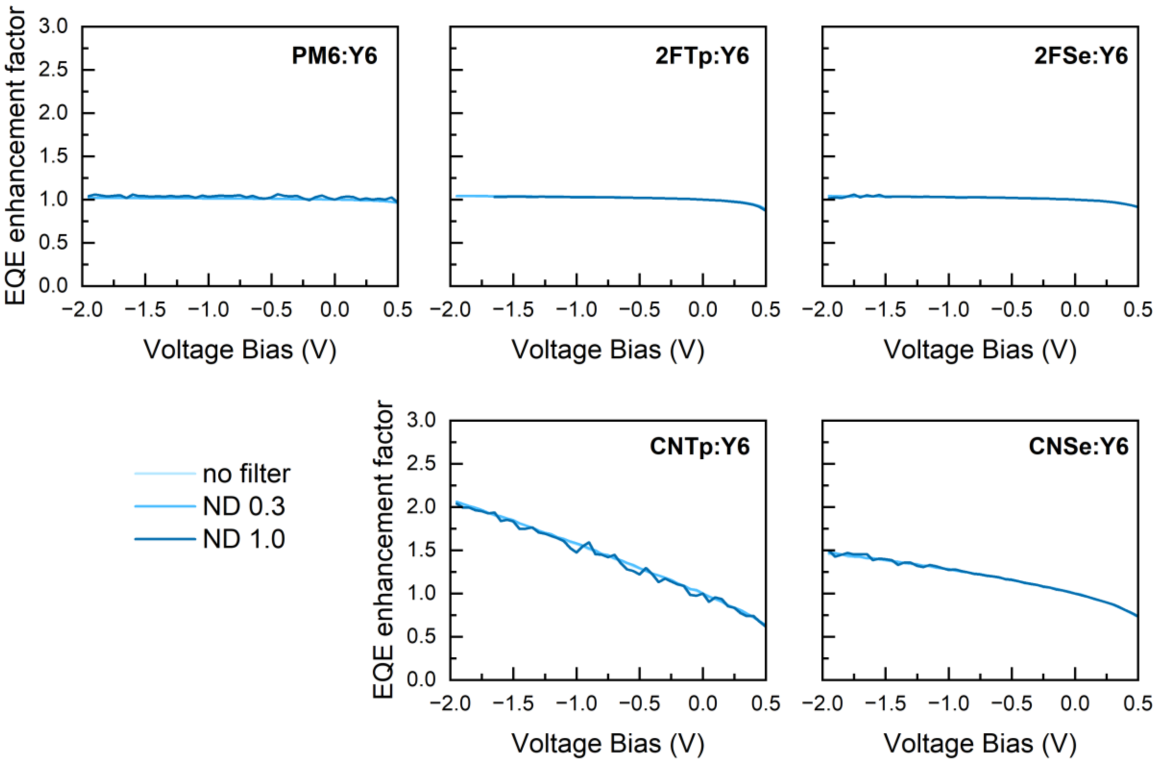

Figure S 17: EQE voltage bias dependence for different intensities of the monochromatic light beam. No difference is observed for the lower illumination source.

8. Charge Carrier Lifetimes

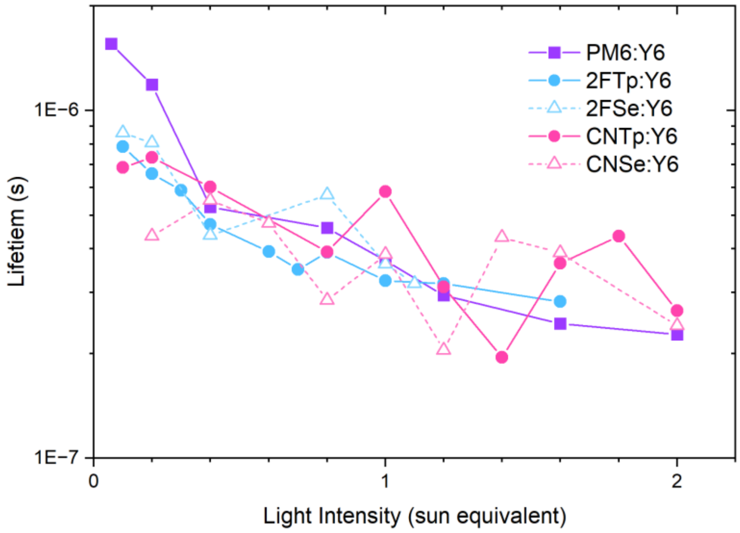

Figure S18: Charge Carrier lifetimes obtained from transient photo-charge (TPQ) measurements at different light intensities of the four devices and the reference. No significant difference in charge carrier lifetimes was observed.

9. Surface Morphology

We used AFM to investigate the surface morphology of the pristine polymer materials (Figure S19), as well as the surface morphology of polymer:Y6 blends (Figure S20).

The depth of the features is rather similar in all films, ranging from 9 – 12 nm in the pristine films and 8 – 16 nm in the blends. Also, root mean square (RMS) values, which is an indication of the surface roughness, are similar 1.1 – 1.6 in the pristine films and from 0.8 – 1.6 in the blends. This means, that in terms of quantitative measures, the surface morphology of for the different polymer films is rather similar. However, judging the images by their qualitative appearance, the both the pristine as well as the blend film with CNSe shows a more fibrous structure in comparison to all the other films. And looking only at the blends, the thiophene films have a more granular feature (little clumps).

We would like to note, that from this similarity in surface morphology, one cannot draw strong conclusions about the internal morphology of the film. More in-depth analysis, like GIWAXS or GISAXS measurements would be necessary to infer more about the internal structure.

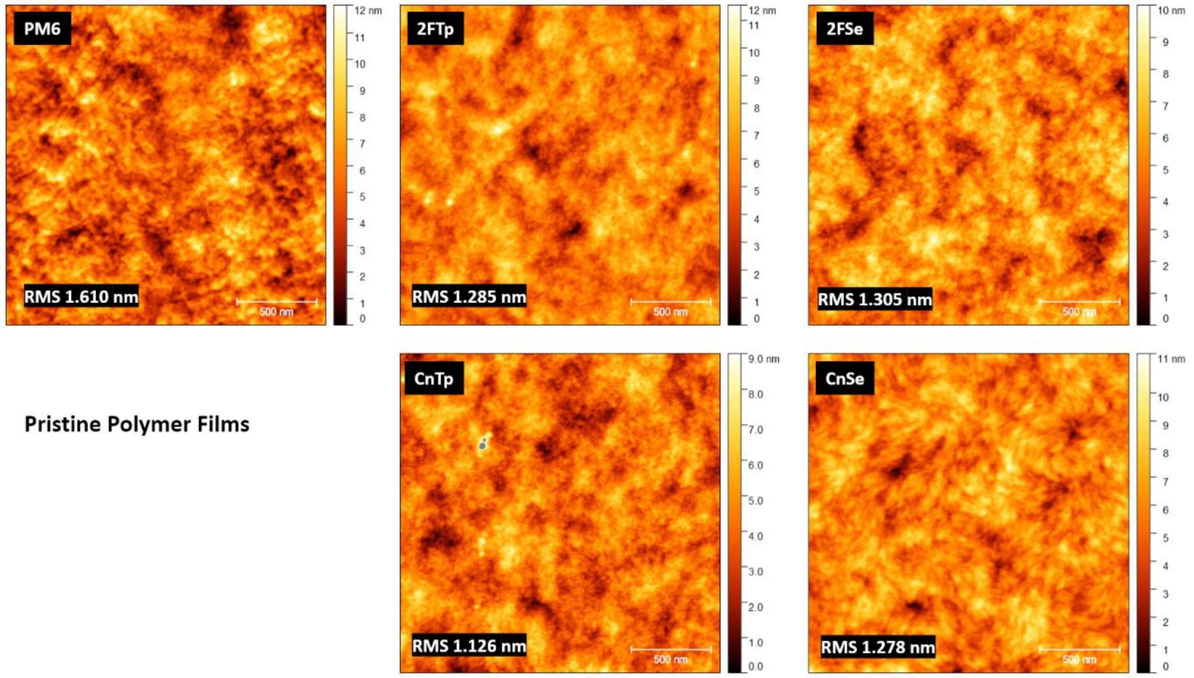

Figure S19: Surface morphology obtained with AFM of pristine films made with the four types of polymers, and PM6 as a reference. The shorthand for the material name and the root mean square are displayed on each AFM image.

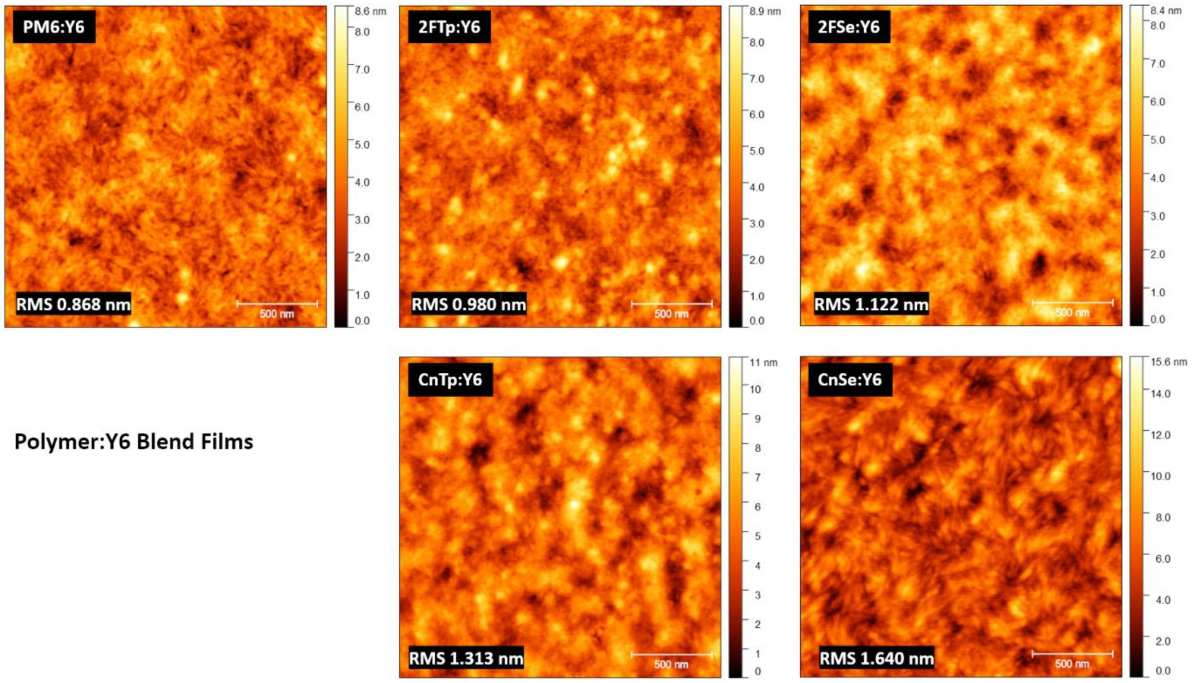

Figure S20: Surface morphology obtained with AFM of blend films with Y6 made with the four types of polymers, and PM6 as a reference. The shorthand for the material name and the root mean square are displayed on each AFM image.

10. SCLC fitting with gpvdm

Table S1: Parameters used in gpvdm fitting and calculated resulting trap densities and effective mobilities.

**gpvdm constant parameters**	
number of free charge carrier density n_f	1e21
effective density of free hole states N_c	1e26 m-3
number of traps	10 bands
gap energy E_g	1.45 eV
and the room temperature energy $k_b T$	25.9 meV

	**gpvdm fitting parameters**			**calculated results**	
**Material**	μ_0 **(cm²V⁻¹s⁻¹)**	N_h **(m⁻³eV⁻¹)**	E_t **(eV)**	n_t **(m⁻³)**	μ^h_{eff}
PM6	1E-7	3.29E23	0.203	1.53731E22	6.10759E-5
2FTp	7.6E-9	1.07E24	0.109	7.56375E21	8.87462E-6
2FSe	6.89E-9	2E24	0.148	3.94722E22	1.7024E-6
CNTp	9.5E-9	6E24	0.178	2.00006E23	4.72623E-7
CNSe	6.67E-9	5E24	0.177	1.64174E23	4.03816E-7
PM6:Y6	3.43E-8	6.24E23	0.15	1.28216E22	2.48162E-5
2FTp:Y6	2.23E-8	1.5E24	0.145	2.78199E22	7.7377E-6
2FSe:Y6	2.36E-8	3.2E24	0.121	3.29373E22	6.95401E-6
CNTp:Y6	2.02E-8	1.8E24	0.158	4.30833E22	4.58223E-6
CNSe:Y6	1.37E-8	2.31E24	0.149	4.65233E22	2.8828E-6

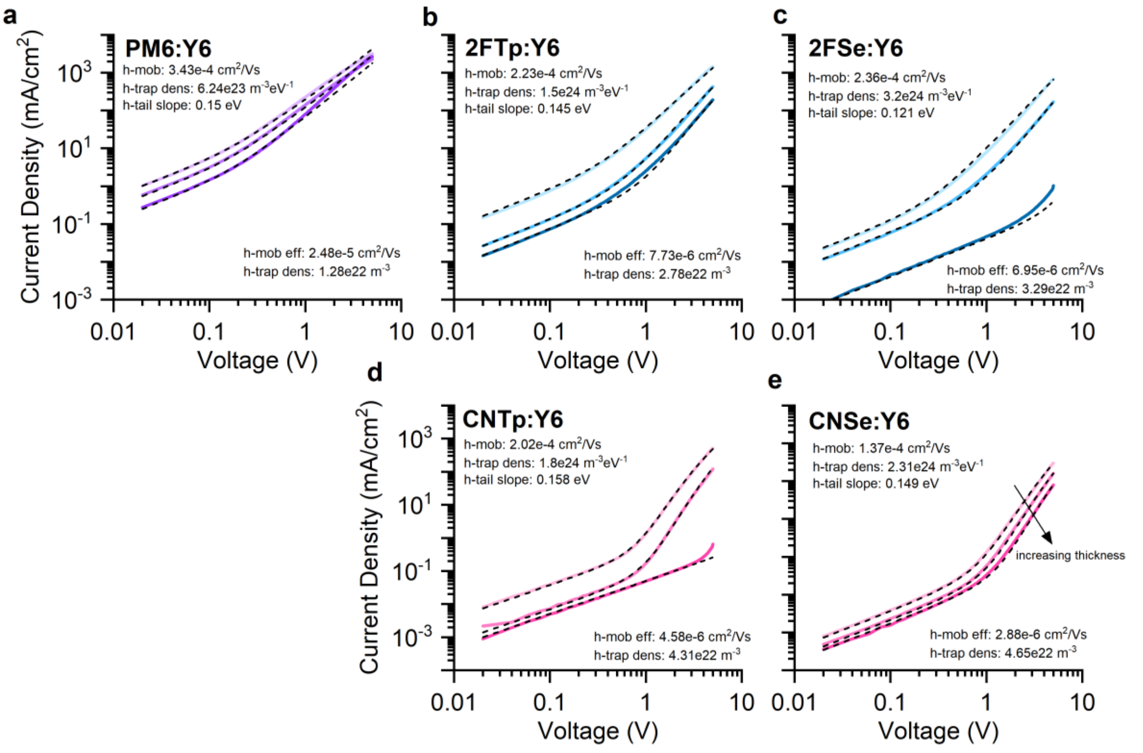

Figure S21: Fitting of the SCLC curves of polymers:Y6 blends with gpvdm as described in the Methods section of the main text. Solid lines are experimental data and dashed lines are fitted. From a-e we show PM6:Y6, 2FTp:Y6, 2FSe:Y6, CNTp:Y6, CNSe:Y6.

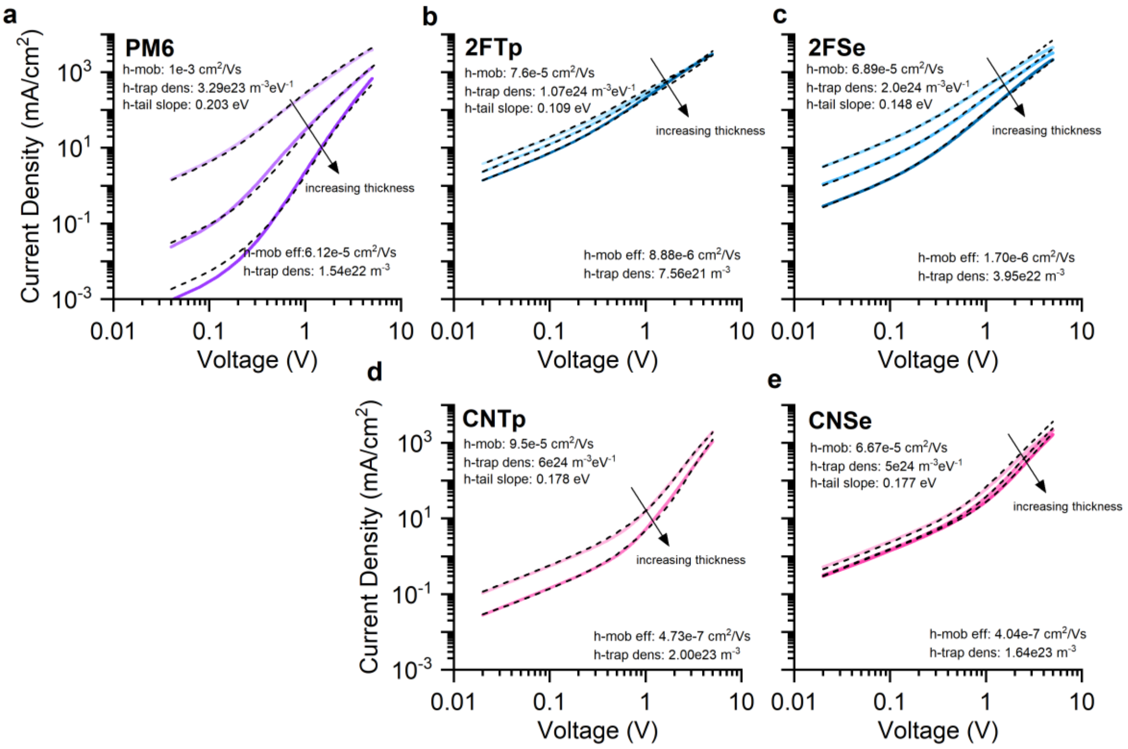

Figure S22: Fitting of the SCLC curves of pristine polymers with gpvdm as described in the Methods section of the main text. Solid lines are experimental data and dashed lines are fitted. From a-e we show PM6, 2FTp, 2FSe, CNTp, CNSe.

11. Numerical Modelling

Here, we show a brief discussion of the different parameters affect the modelling results individually or in selected subsets. All model parameters and fitting parameters are shown in Table S2 and Table S3.

Lowering the offset $\Delta E_{\mathrm{LE-CT}}$ (between the exciton and the CT state) increases the V_{oc} and lowers the PL quenching due to increased CT to exciton reformation $k^{\mathrm{ref}}_{\mathrm{CT\rightarrow LE}}$, while the J_{sc} remains unchanged (Figure S23a). It is also expected that with a smaller offset the exciton dissociation rate is decreased, however, in this model, the dissociation rates are not automatically calculated from the offset, but rather controlled explicitly through the parameters $k^{\mathrm{dis}}_{\mathrm{LE\rightarrow CT}}$ and $k^{\mathrm{dis}}_{\mathrm{CT\rightarrow CS}}$. Decreasing the exciton dissociation alongside the offset predominantly increases the absolute PL intensity, because fewer excitons dissociate and instead recombine radiatively to ground (Figure S23b). At the same time the charge generation (and thus the J_{sc}) remains mostly unaffected, as long as the exciton dissociation is still faster than the CT dissociation rate (Figure S23b). To reduce the charge generation efficiency, it is indeed necessary to additionally reduce the $k^{\mathrm{dis}}_{\mathrm{CT\rightarrow CS}}$, which strongly reduces the J_{sc} while having only minor impacts on the PL quenching (Figure S23c).

Without consideration of the exciton dissociation, it was not possible to reproduce the trends observed in the PL quenching, and without considering the CT dissociation there is no sufficient reduction in the J_{sc}. Alternatively, when trying to tune only the offset and the mobility, a rough agreement between the model and the experimental JV and PL was achieved, however, the required changes in the mobility are much larger than in the experimentally observed mobilities. In addition, the difference between the PL emission of the 2FSe and the 2FTp cannot be explained with the mobility alone (Figure S23d).

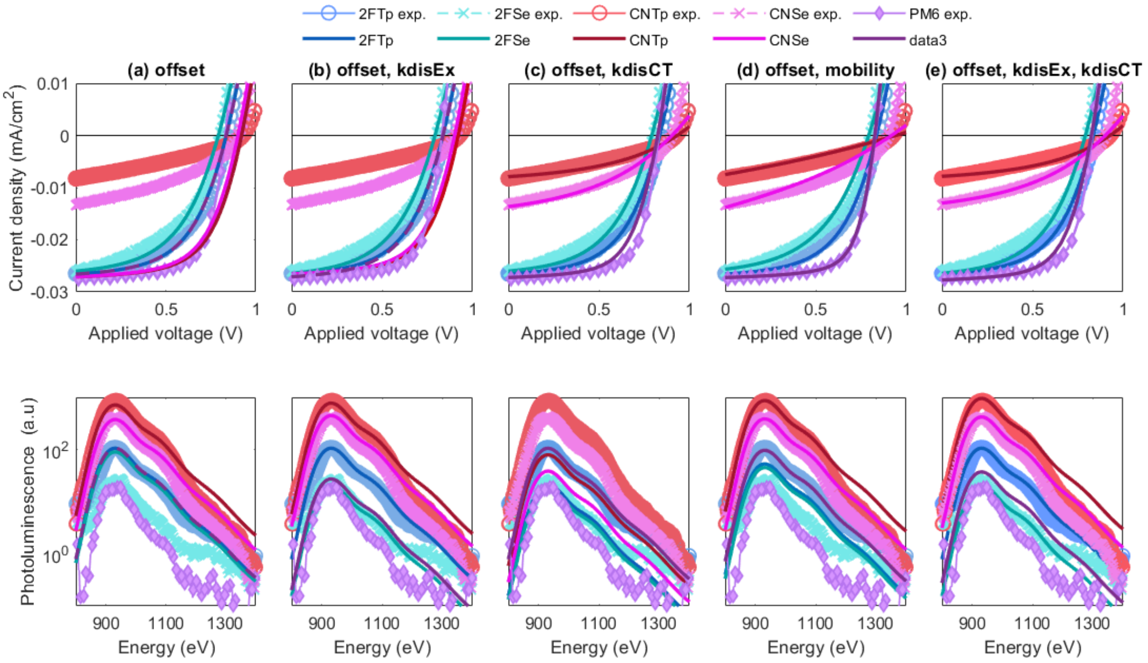

Figure S23: Fitting of the JV and PL data using different parameter restrictions.

Table S2:Fixed Model Parameters

Parameter	Value	Unit
Active Layer Thickness	100	nm
CS state energy	1.35	eV
LE oscillator strength	2.56	/
CT oscillator strength	1e-4	/
LE sigma	0.01	/
CT sigma	0.01	/
LE number of states	1	/
CT number of states	1	/
Ratio LE to CT states	0.1	/
Exciton density	3e27	m^{-3}
LE reorganisation energy (outer)	0.075	eV
LE reorganisation energy (inner)	0.05	eV
LE reorganisation energy (outer)	0.07	eV
LE reorganisation energy (inner)	0.1	eV
Number of charge carriers	1e18	/
Bfor	5e-11	cm^3/s

Table S3: Input parameters for the fits shown in Figure S23. Parameters that were deliberately kept constant are shaded with a grey background. The parameters that are free to change (apart from the offset which is always allowed to change) are shown in the legend on the left.

		Input Parameters				Output Values		
**Offset**		**Offset**	**kdisLE**	**kdisCT**	**mobility**	**Jsc**	**Voc**	**FF**
	2FTp:Y6	0.25	1e10	1e10	6e-5	26.59	0.83	0.53
	2FSe:Y6	0.29	1e10	1e10	6e-5	26.01	0.79	0.49
	CNTp:Y6	0.27	1e10	1e10	6e-5	27.13	0.92	0.59
	CNSe:Y6	0.19	1e10	1e10	6e-5	27.09	0.90	0.59
	PM6:Y6	0.25	1e10	1e10	6e-5	26.59	0.83	0.53
**Offset, k_{dis-LE}**		**Offset**	**kdisLE**	**kdisCT**	**mobility**	**Jsc**	**Voc**	**FF**
	2FTp:Y6	0.25	1e10	1e10	6e-5	26.59	0.84	0.53
	2FSe:Y6	0.29	5e10	1e10	6e-5	26.44	0.79	0.50
	CNTp:Y6	0.21	5e9	1e10	6e-5	26.59	0.92	0.59
	CNSe:Y6	0.23	5e9	1e10	6e-5	26.55	0.90	0.59
	PM6:Y6	0.25	10e10	1e10	6e-5	27.08	0.84	0.53
**Offset, k_{dis-CT}**		**Offset**	**kdisLE**	**kdisCT**	**mobility**	**Jsc**	**Voc**	**FF**
	2FTp:Y6	0.25	1e10	1e10	6e-5	26.58	0.84	0.53
	2FSe:Y6	0.29	1e10	1e10	6e-5	26.01	0.79	0.50
	CNTp:Y6	0.21	1e10	4e7	6e-5	7.90	0.93	0.41
	CNSe:Y6	0.23	1e10	1.5e8	6e-5	13.64	0.90	0.39
	PM6:Y6	0.23	1e10	5e10	6e-5	27.24	0.82	0.61
**Offset, mobility**		**Offset**	**kdisLE**	**kdisCT**	**mobility**	**Jsc**	**Voc**	**FF**
	2FTp:Y6	0.25	1e10	1e10	6e-5	26.59	0.84	0.53
	2FSe:Y6	0.29	1e10	1e10	5e-5	25.64	0.79	0.47
	CNTp:Y6	0.21	1e10	1e10	2e-6	7.50	0.94	0.28
	CNSe:Y6	0.23	1e10	1e10	5e-5	13.81	0.92	0.31
	PM6:Y6	0.25	1e10	1e10	2e-4	27.24	0.83	0.67
**Offset, k_{dis-LE}, k_{dis-CT}**		**Offset**	**kdisLE**	**kdisCT**	**mobility**	**Jsc**	**Voc**	**FF**
	2FTp:Y6	0.25	1e10	1e10	6e-5	26.59	0.84	0.53
	2FSe:Y6	0.29	5e10	1e10	6e-5	26.44	0.79	0.50
	CNTp:Y6	0.21	1e9	5e7	6e-5	7.83	0.93	0.42
	CNSe:Y6	0.23	2.5e9	1.5e8	6e-5	12.97	0.90	0.39
	PM6:Y6	0.23	5e10	5e10	6e-5	27.70	0.83	0.61
